# Non‐Invasively Tracking an Arbitrary Deformable Object Through Scattering Media and Around Corners via Speckle Correlations

**DOI:** 10.1002/advs.75072

**Published:** 2026-04-03

**Authors:** Aiping Zhai, Wenjing Ji, Yan Wang, Xiyuan Luo, Wenjing Zhao, Dong Wang, Fei Liu

**Affiliations:** ^1^ College of Physics and Optoelectronics Engineering Taiyuan University of Technology Yingze P. R. China; ^2^ School of Optoelectronic Engineering Xidian University Xi'an P. R. China; ^3^ Shanxi Key Laboratory of Precision Measurement Physics Taiyuan University of Technology Taiyuan P. R. China; ^4^ Key Laboratory of Advanced Transducers and Intelligent Control System Ministry of Education and Shanxi Province Taiyuan University of Technology Yingze P. R. China

**Keywords:** a moving deformable target, around corners, non‐invasive tracking, scattering media

## Abstract

Tracking a hidden object is of great significance across various fields. Although several methods have attempted to track an object behind scattering media, they either only work by assuming the object's shape is invariant, or require prior information. Here, we propose a speckle correlation centroid localization method (SCCLM) for non‐invasively tracking a moving deformable object behind or inside scattering media and around corners, without any prior information. After capturing speckle patterns of the object at different moments, the relative centroid displacements can be determined by locating the centroids of speckle auto‐correlation (SAC) and speckle cross‐correlation (SCC) at the adjacent moments, rather than conventionally locating the peak positions of SAC and SCC. The trajectory of the object is then reconstructed by sequentially superimposing the relative centroid displacements. Experimental results validate that the SCCLM can track a hidden object moving beyond the memory effect region, which is not only scaling, rotating, and stretching, but also completely changing into different shapes. Furthermore, its effectiveness is verified for tracking a moving deformable object through dynamic scattering media, such as chicken breast tissue, and around corners. Even when the scene grows more complex with many objects, the SCCLM remains valid.

## Graphical abstract

A speckle correlation centroid localization method (SCCLM) is proposed for non‐invasive tracking of an arbitrary deformable object through scattering media and around corners, requiring no prior information. Its effectiveness is demonstrated not only in tracking hidden objects beyond the memory effect region but also in following objects undergoing scaling, rotation, stretching, and even complete shape changes.

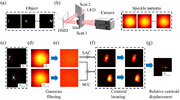

## 1 Introduction

Inhomogeneous scattering media, such as biological tissues, smog, and turbid water, scramble the light path, causing the image to be blurred or distorted as a noise‐like speckle pattern [1, 2]. In such a case, directly observing the trajectory of a moving object behind or inside scattering media is challenging [3]. Recently, many methods have been proposed to exploit scattered light. For instance, methods reliant on iterative wavefront shaping techniques [4, 5, 6, 7, 8, 9] and transmission matrix measurement [10, 11, 12, 13]. However, they require the necessary detection on both sides of a scattering medium. The deep learning methods enable imaging or tracking a moving object through scattering media [14, 15], while the invasive acquisition of large training datasets is unavoidable.

A possible solution is based on persistent correlations of the scattering light, which does not depend on the details of the media [16, 17, 18, 19, 20]. Speckle patterns formed by the incident light shining from different angles are mutually correlated within the region of the memory effect (ME), which contains information about the shapes and positions of the object [21, 22, 23, 24]. Recently, methods including utilizing the correlations of speckle patterns and optimizing the optical transfer function have been proposed to track an object behind or inside scattering media non‐invasively [25, 26, 27, 28]. Compared with deep learning approaches, less computational effort is required, but they are only available for tracking an object, assuming its shape is invariable [25, 26] or requiring prior information [27, 28]. This is unrealistic in practice since there is normally no prior information, as well as the relative orientation and/or distance between the object and detector always changes randomly, making the observed shape of the moving object deform accordingly. Thus far, how to non‐invasively track a moving deformable object behind or inside scattering media and around corners without prior information remains an open and challenging issue.

Within ME, a scattering imaging system can be considered a linear shift‐invariant system [29, 30]. As a result, the relative displacements of a moving object can be calculated by exploring the correlations of the speckle patterns. Therefore, researchers proposed to calculate the peak positions [17, 18, 25, 27, 31, 32, 33, 34, 35] of speckle auto‐correlation (SAC) and speckle cross‐correlation (SCC) at adjacent moments for tracking a moving object behind scattering media. Indeed, the methods are effective when the object is only translated during motion (See Figure S1). However, once the shape of the moving object is changed, such as rotation with translation, scaling with translation, or even complete deformation, the existing methods mentioned above probably fail (See Figures S2–S4).

Fortunately, we find that the relative centroid displacements of the moving deformable object at adjacent moments can be determined by locating the centroids of SAC and SCC, and for the first time, we explicitly give a general relationship that the spatial location of the object cross‐correlation centroid equals that of the corresponding speckle cross‐correlation centroid (see Equation 1 and Section S9). Thanks to this finding, a speckle correlation centroid localization method (SCCLM) is proposed, which directly utilizes the speckle patterns, without any prior information, to non‐invasively track a moving deformable object behind or inside scattering media, and around corners. We demonstrate, theoretically and experimentally, that by locating the centroids of SAC and SCC, the relative centroid displacements of the moving deformable object can be determined. Then, the trajectory of the object can be obtained via sequentially superimposing the relative centroid displacements of the object at different moments, although the moving range of the object exceeds the ME region. Experimental verifications show that the trajectory of the moving object can be effectively reconstructed, while it is hidden behind not only static scattering media such as single and double layers of ground glass diffuser but also dynamic scattering media with strong decorrelation such as chicken breast tissue, inside the scattering media and around corners, even complex scenes with many static objects, verifying the effectiveness of the proposed method.

## 2 Results

### 2.1 Principle of SCCLM

**FIGURE 1:**
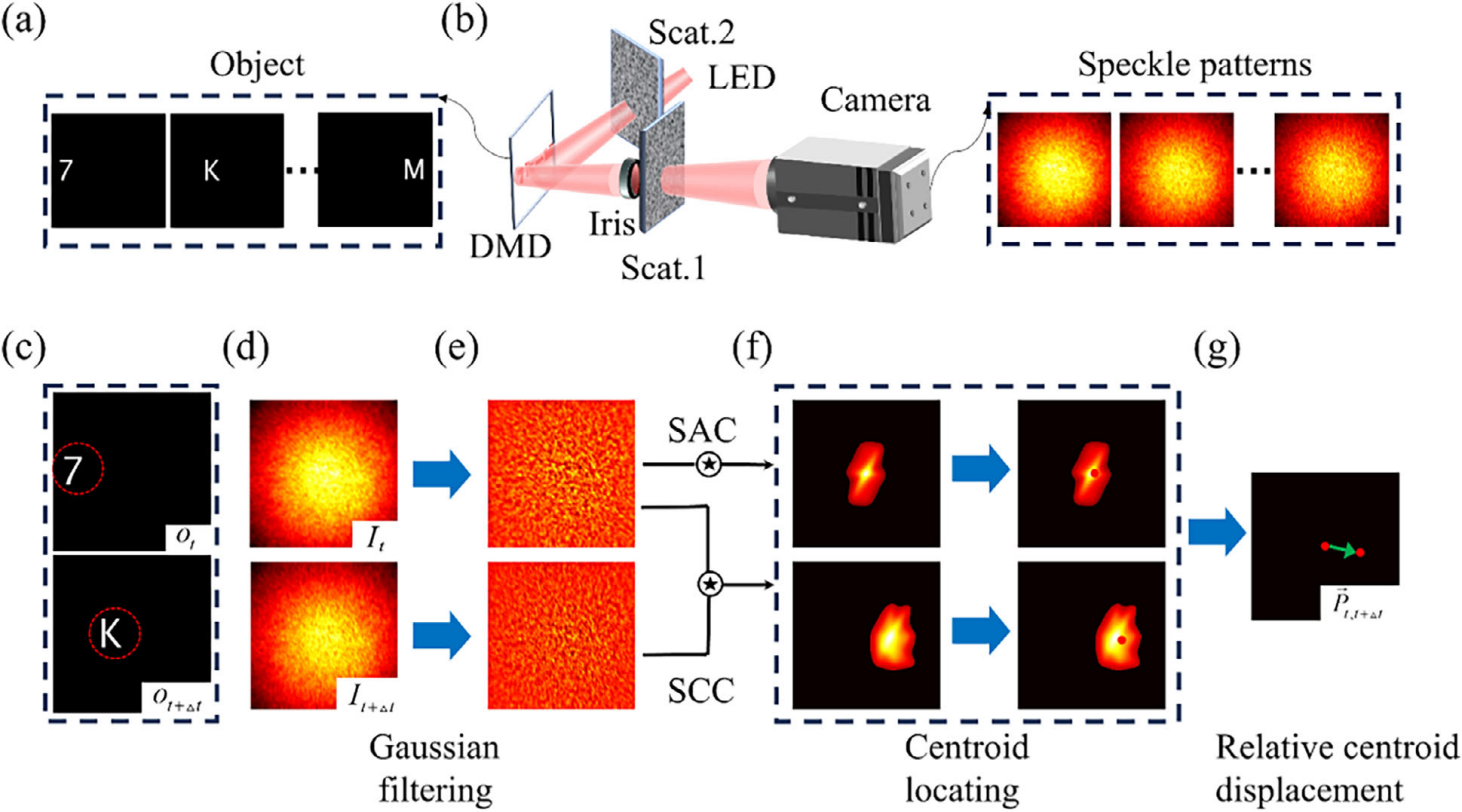
Non‐invasively tracking a moving deformable object utilizing the SCCLM. A moving deformable object behind or inside scattering media (a) is simulated on the DMD, and the speckle patterns are captured by the camera (b). (c) The moving object changes into a different shape from the time *t* to *t* + ▵*t*, and the red‐dashed circles represent the ME region. (d) The corresponding speckle patterns. (e) The Gaussian‐filtered speckle patterns. (f) Locating the centroids of the auto‐correlation (top) and cross‐correlation (bottom) of the filtered speckle patterns, and marking them with red dots. (g) The relative centroid displacement of the object in the image plane is indicated by a green arrow.

Assuming a moving object, hidden behind or inside scattering media and around corners, is deforming during the time of *t* and *t* + ▵*t*, denoted as *O_t_* and *O*_*t* + ▵*t*_, as shown in Figure 1c. Light emitted or reflected from the moving object is randomly scattered and produces speckle patterns *I_t_* and *I*_*t* + ▵*t*_, respectively (Figure 1d), which are collected by a camera. Such a process can be expressed as the convolution of the ideal images of the object and the point spread function (PSF), e.g., *I_t_* (*u*,*v*) = *O_t_* (*u*,*v*)*PSF_t_ (*u*,*v*), where *u* and *v* are spatial coordinates in the image plane, * denotes the convolution operation, *PSF*_*t* + ▵*t*_(*u*, *v*) is the PSF corresponding to the ME region, in which the object *O*_*t* + ▵*t*_ is located. During the time interval ▵*t*, the adjacent ME regions, in which the object is moving and deforming, have enough overlap, which guarantees*PSF_t_* (*u*,*v*) ≈ *PSF*_*t* + ▵*t*_(*u*, *v*). As a result, the cross‐correlation between *PSF_t_* (*u*,*v*) and *PSF*_*t* + ▵*t*_(*u*, *v*) can be expressed as δ(*u*, *v*). The correlation between speckle pattern *I_t_* and speckle pattern *I*_*t* + ▵*t*_ (i.e., SCC) can be expressed as:

(1) $$\begin{array}{ccc} I_{t}\left(u,v\right)★I_{t+▵t}\left(u,v\right) & = & \left[O_{t}\left(u,v\right)*PSF_{t}\left(u,v\right)\right]\\ & & ★\left[O_{t+▵t}\left(u,v\right)*PSF_{t+▵t}\left(u,v\right)\right]\\ & = & \left[O_{t}\left(u,v\right)★O_{t+▵t}\left(u,v\right)\right]\\ & & *\left[PSF_{t}\left(u,v\right)★PSF_{t+▵t}\left(u,v\right)\right]\\ & = & O_{t}\left(u,v\right)★O_{t+▵t}\left(u,v\right)*\delta \left(u,v\right)\\ & = & O_{t}\left(u,v\right)★O_{t+▵t}\left(u,v\right)+B \end{array}$$

where ⋆ represents the correlation calculation, *B* represents an additional constant background term from the speckle cross‐correlation (See Section S9 for more details), which can be eliminated from the speckle cross‐correlation image by applying a thresholding procedure. The ideal images *O_t_* (*u*,*v*) and *O*_*t* + ▵*t*_(*u*, *v*), of the object, deformed at the adjacent moment, can be re‐expressed in terms of shape functions and centroid coordinates:

(2) $$\left\{\begin{array}{c} O_{t}\left(u,v\right)=S_{t}(u-{\bar{u}}_{t},v-{\bar{v}}_{t})\\ O_{t+▵t}\left(u,v\right)=S_{t+▵t}(u-{\bar{u}}_{t+▵t},v-{\bar{v}}_{t+▵t}) \end{array}\right.$$

where *S_t_* (*u*,*v*) and *S*_*t* + ▵*t*_(*u*,*v*) denote the shape functions at *t* and *t* + ▵*t*, $\left({\bar{u}}_{t},{\bar{v}}_{t}\right)$ and $\left({\bar{u}}_{t+▵t},{\bar{v}}_{t+▵t}\right)$ represent the centroid coordinates, respectively.

By substituting Equation 2 into Equation 3, *O_t_* (*u*,*v*)⋆*O*_*t* + ▵*t*_(*u*, *v*) can be re‐expressed as:

(3) $$\begin{array}{ccc} O_{t}\left(u,v\right)★O_{t+▵t}\left(u,v\right) & = & S_{t}(u-{\bar{u}}_{t},v-{\bar{v}}_{t})\\ & & ★\;S_{t+▵t}(u-{\bar{u}}_{t+▵t},v-{\bar{v}}_{t+▵t})\\ & = & \;\int \int \;S_{t}(u-{\bar{u}}_{t},v-{\bar{v}}_{t})\cdot S_{t+▵t}\left(u+p\right.\\ & & -\left.{\bar{u}}_{t+▵t},v+q-{\bar{v}}_{t+▵t}\right)dudv \end{array}$$

Let $u=u^{\prime }+{\bar{u}}_{t},v=v^{\prime }+{\bar{v}}_{t}$,

(4) $$\begin{array}{ccc} O_{t}\left(u,v\right)★O_{t+▵t}\left(u,v\right) & = & \;\;\;\int \int \;S_{t}(u^{\prime },v^{\prime })\cdot S_{t+▵t}\left(u^{\prime }+p\right.\\ & & -\left.\left({\bar{u}}_{t+▵t}-{\bar{u}}_{t}\right),v^{\prime }\;+\;q\;-\;\left({\bar{v}}_{t+▵t}-{\bar{v}}_{t}\right)\right)du^{\prime }dv^{\prime }\\ & = & C\left(p-\left({\bar{u}}_{t+▵t}-{\bar{u}}_{t}\right),q-\left({\bar{v}}_{t+▵t}-{\bar{v}}_{t}\right)\right) \end{array}$$

where *C*(*p*, *q*) denotes the cross‐correlation function between the shape functions *S_t_* (*u*,*v*) and *S*_*t* + ▵*t*_(*u*,*v*).

By substituting Equation 4 into Equation 5, it can be re‐expressed as:

(5) $$I_{t}\left(u,v\right)★I_{t+▵t}\left(u,v\right)-B=C(p-\left({\bar{u}}_{t+▵t}-{\bar{u}}_{t}\right),q-({\bar{v}}_{t+▵t}-{\bar{v}}_{t}))$$

Therefore, according to the definition of centroid [36]*^,^* by the centroids of *I_t_* (*u*,*v*)⋆*I_t_* (*u*,*v*) and *I_t_* (*u*,*v*)⋆*I*_*t* + ▵*t*_(*u*, *v*), as shown in Figure 1f, the relative centroid displacement ${\vec{P}}_{t,t+▵t}$ of the moving object between the times of *t* and *t* + ▵*t* (as shown in Figure 1g) can be calculated as:

(6) $$\begin{array}{ccc} {\vec{P}}_{t,t+▵t} & = & centroid\left[I_{t}\left(u,v\right)★I_{t+▵t}\left(u,v\right)-B\right]\\ & & -\;centroid\left[I_{t}\left(u,v\right)★I_{t}\left(u,v\right)-B\right]\;\\ & = & centroid[C(p-\left({\bar{u}}_{t+▵t}-{\bar{u}}_{t}\right),q-({\bar{v}}_{t+▵t}-{\bar{v}}_{t}))]\\ & & -\;centroid[C(p,q)]\;=\left({\bar{u}}_{t+▵t}-{\bar{u}}_{t},{\bar{v}}_{t+▵t}-{\bar{v}}_{t}\right) \end{array}$$

In brief, working with Equation 6, the relative orientations and positions of the moving deformable object at different moments can be calculated with the collected speckle patterns directly, without the need for estimated fingerprints [30, 31], or PSFs [17, 18, 37]. Then, the trajectory of the moving object, hidden behind or inside scattering media and around corners, can be determined.

When the hidden object undergoes only translation without shape changing, the SCC*I_t_* (*u*,*v*)⋆*I*_*t* + ▵*t*_(*u*, *v*) is centrally symmetric (See Section S9 for more details), and the peak position of the SCC happens to coincide with its centroid position. This suggests the existing methods (locating the peak position of the SCC) [17, 18, 25, 27, 31, 32, 33, 34, 35] is only a special case, while our SCCLM, which locates the centroid of SCC, is a general method.

### 2.2 Experimental Setup

To validate the SCCLM experimentally, two different setups for tracking a moving deformable object behind or inside scattering media are built. To simulate a scene where the object is behind scattering media, we generated a moving deformable object via a digital micromirror device (DMD, DLP4500, 912 × 1140 pixels), and illuminated it with a 625 nm light‐emitting diode (LED, Thorlabs, M625L4), as shown in Figure S5a. The light reflected by the moving object at the different moments passes through a 4 mm iris and scattering media sequentially, forming speckle patterns captured by the camera (Andor Zyla 5.5, 2560 × 2160 pixels, pixel size 6.5 µm). Here, the iris is used to adjust the size of the speckle grains, which is regarded here as a major factor that determines the tracking accuracy. The distances from the scattering media to the object and camera are denoted by *u* and *v*, which are set as 105 mm and 75 mm, respectively, to satisfy the Nyquist sampling requirement for the speckle patterns.

To simulate a scene where the object is inside scattering media, the only difference made is that a scattering medium was added between the LED and DMD, as shown in Figure 1b and Figure S5b. The rest of the settings are the same as in Figure S5a. In the experiment, the LED light irradiates scattering medium 2 with a spot diameter of 4 mm. Then, the scattered light propagates 80 mm before illuminating the DMD. The size of the moving deformable object simulated by DMD is about 40–60 DMD pixels. In addition, the beam illumination diameter on scattering medium 1 is about 4 mm.

### 2.3 Tracking a Moving Deformable Object Behind a Single‐Layer Ground Glass Diffuser

**FIGURE 2:**
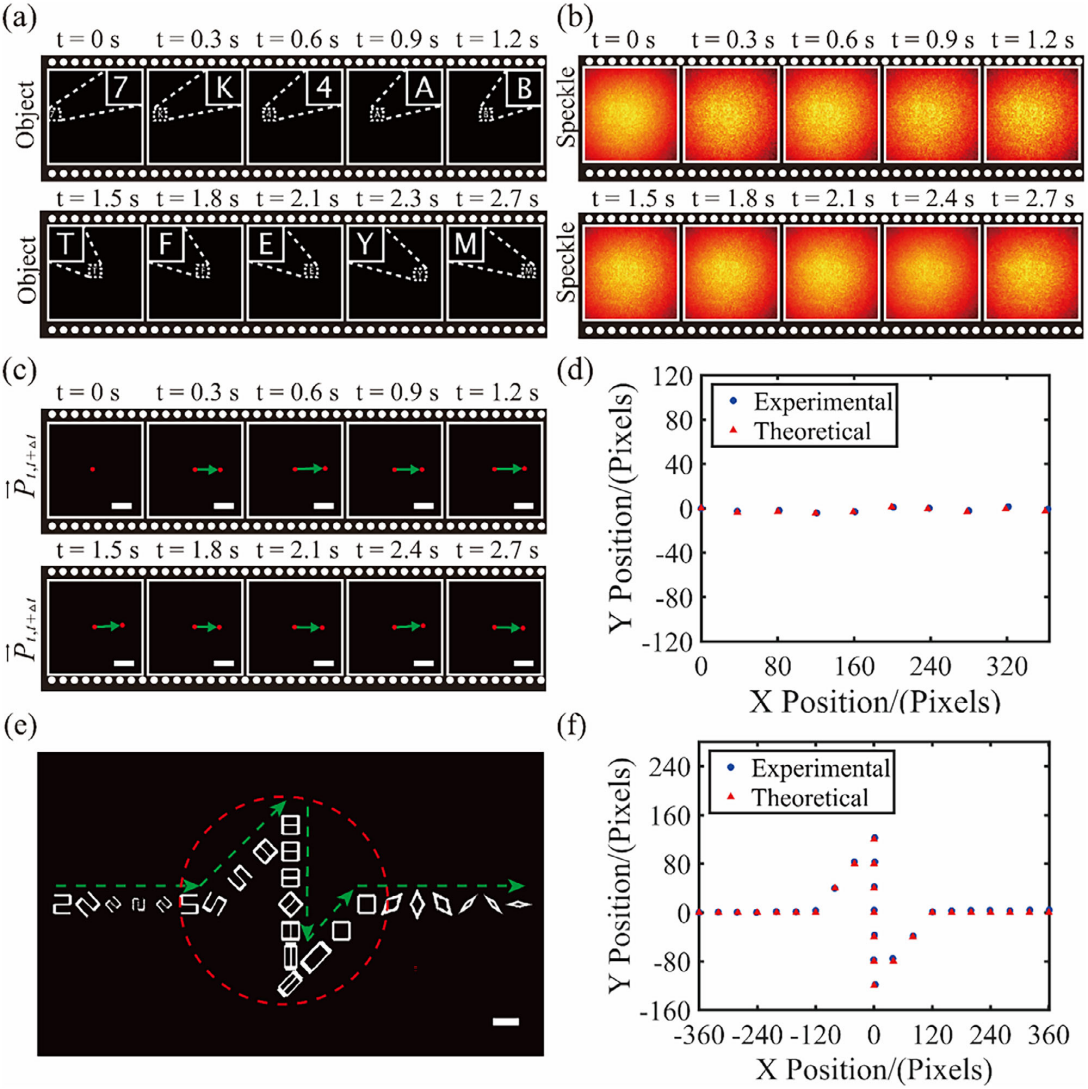
Tracking a moving deformable object behind a single‐layer ground glass diffuser. (a) Ground truth. An object, hidden behind a single‐layer ground glass diffuser, deforms into completely different shapes during motion. (b) The speckle patterns captured by the camera. (c) The relative centroid displacements of the moving object in the image plane. Scale bar: 20 camera pixels. (d) The comparison of the theoretical trajectory (red triangles) of the object in (a) and the trajectory reconstructed by the SCCLM (blue dots). (e) A deforming object with arbitrary trajectory changes, hidden behind a single‐layer ground glass diffuser. The green arrows indicate the moving direction, and the red‐dashed circle represents the ME region. Scale bar: 40 DMD pixels. (f) The comparison of the theoretical trajectory (red triangles) of the object in (e) and the trajectory reconstructed by the SCCLM (blue dots).

To prove the concept, the SCCLM is first employed to track a moving deformable object behind a single‐layer ground glass diffuser. In this experiment, the camera exposure time was set to 0.3 s, a value determined by two conditions. The first condition concerns the temporal decorrelation properties of the scattering medium. The second ensures that the object displacement between adjacent frames remains within the ME range. and applied to for all subsequent experiments. The time intervals for the subsequent experiments were chosen following the same two criteria, although different kinds of scattering enviroments were investigated. The moving object is simulated to be not only scaling, rotating, and stretching (Figure S6), but also deforming into completely different shapes (Figure 2a). Different objects are displayed on the DMD in turn to simulate a moving and deformable object. The deformation of the moving object happens at a time interval of ▵*t* = 0.3 s, and the total moving distance is 2.7 times the ME region. The light emitted from the moving object at the different moments propagates through a ground glass diffuser (DG10‐120, Thorlabs) (the unilateral ME region is approximately 135 DMD pixels), and produces a series of speckle patterns, as shown in Figure 2b [34]. The relative centroid displacements ${\vec{P}}_{t,t+▵t}$ of the moving object in the image plane can be determined by Equation 6, as shown in the green arrows in Figure 2c. Then, the trajectory of the deforming object during motion can be reconstructed by sequentially superimposing the determined relative centroid displacements in the object plane ${\vec{R}}_{t,t+▵t}$, denoted by the blue dots in Figure 2d. The experimental results indicate that the reconstructed trajectory 99.3% overlaps with the theoretical one (the red triangles in Figure 2d), demonstrating the effectiveness of the SCCLM in tracking a moving deformable object, while not limited by the ME region.

Further, we explore the ability of the SCCLM to track a deforming object behind scattering media with arbitrary trajectory changes, as shown in Figure 2e. As before, the trajectory of the object is successfully reconstructed from the speckle patterns utilizing the SCCLM, as shown in Figure 2f (See Video S1). Evidently, the reconstructed trajectory tends to be consistent with the theoretical one, suggesting the capability of the SCCLM in tracking a moving deformable object behind a scattering medium, no matter how it deforms and the trajectory changes.

### 2.4 Tracking a Moving Deformable Object Behind the Chicken Breast Tissue

**FIGURE 3:**
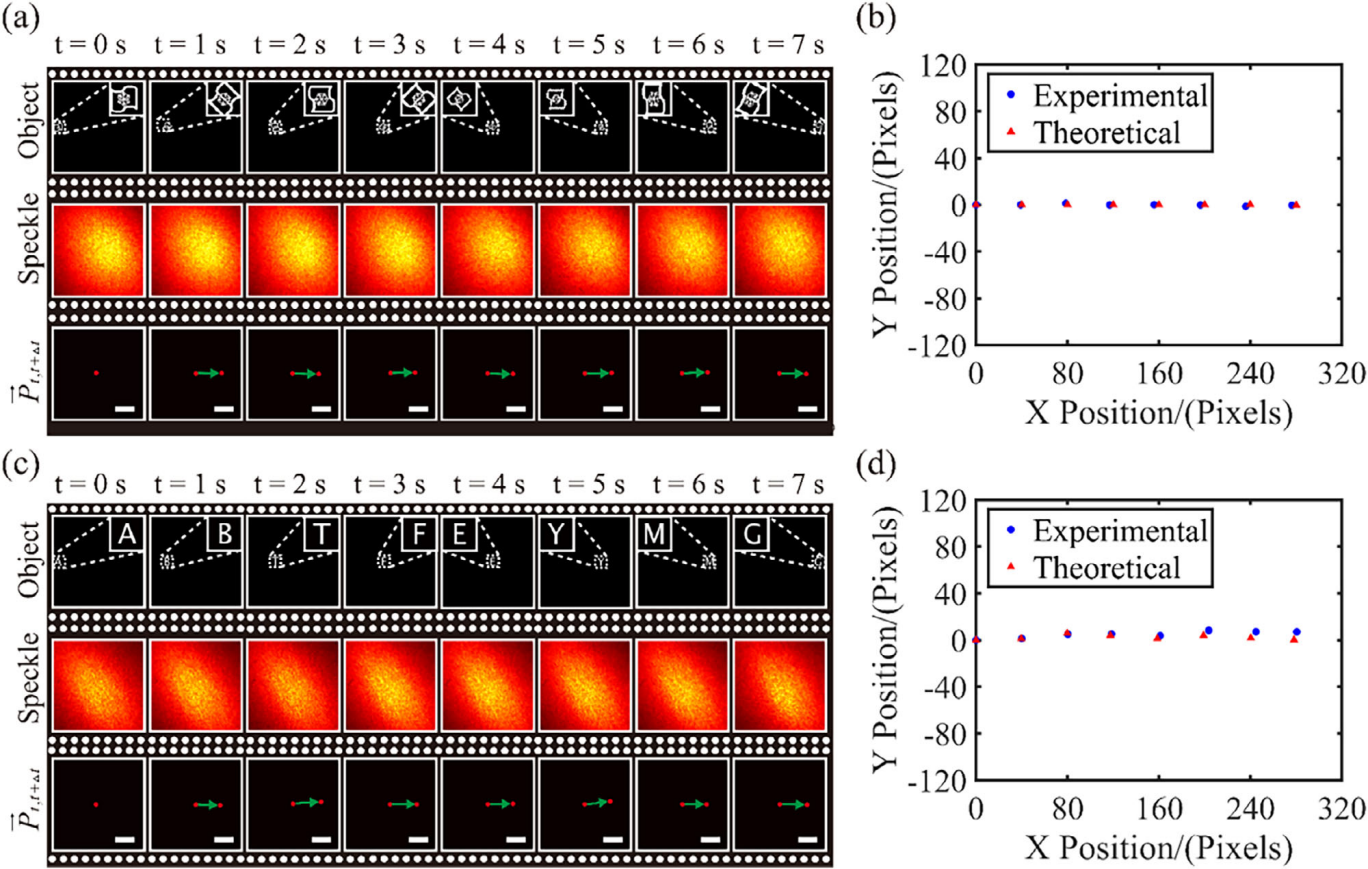
Tracking a moving deformable object behind the chicken breast tissue. (a) and (c) A moving deformable object, hidden behind the chicken breast tissue. The second rows represent the speckle patterns captured by the camera, and the third rows represent the relative centroid displacements of the moving object in the image plane. (b) and (d) The comparison of the reconstructed trajectory (blue dots) and the theoretical trajectory (red triangles) of the objects in (a) and (c), respectively. Scale bar: 20 camera pixels.

The key requirement for using the SCCLM to track a moving deformable object behind chicken breast tissue depends on the correlation of speckle patterns at the adjacent moments. In this experiment, the camera exposure time was set to 1.0 s. The decorrelation in dynamic scattering media makes it more challenging. To further verify the robustness of the SCCLM, we conducted experiments utilizing a 1.2 mm‐thick slice of chicken breast tissue as dynamic scattering media, with a unilateral ME region of 46 DMD pixels. It has been reported that the speckle decorrelation time is 3.4 s for 5 mm thick chicken tissue, and it will be prolonged with the decrease of the slice thickness [38, 39]. Therefore, the time interval of ▵*t* = 1 s was set to guarantee the correlation of speckle patterns captured at the adjacent moments. The first rows of Figure 3a,c show the simulated moving deformable objects. With the proposed SCCLM, the relative centroid displacements ${\vec{P}}_{t,t+▵t}$ of the moving deformable objects in the image plane (third rows of Figure 3a,c) were successfully recovered from the captured speckle patterns. Even if the object moves 280 DMD pixels from the initial position, the reconstruction of the trajectories is successfully achieved by sequentially superimposing the recovered relative centroid displacements of the object plane ${\vec{R}}_{t,t+▵t}$, as depicted in Figure 3b,d, respectively. The reconstructed trajectory is consistent with the theoretical one. The tiny amount of deviations may be from reduced moisture content within the chicken breast tissue, which may affect the scattering properties of the light in the chicken breast tissue and even reduce the correlation between speckle patterns. The results suggest that the tracking of a moving deformable object through dynamic scattering media can be achieved, as long as the correlation is satisfied between adjacent speckle patterns, without being limited by the ME range (See Video S2).

### 2.5 Tracking a Moving Deformable Object Inside Scattering Media

**FIGURE 4:**
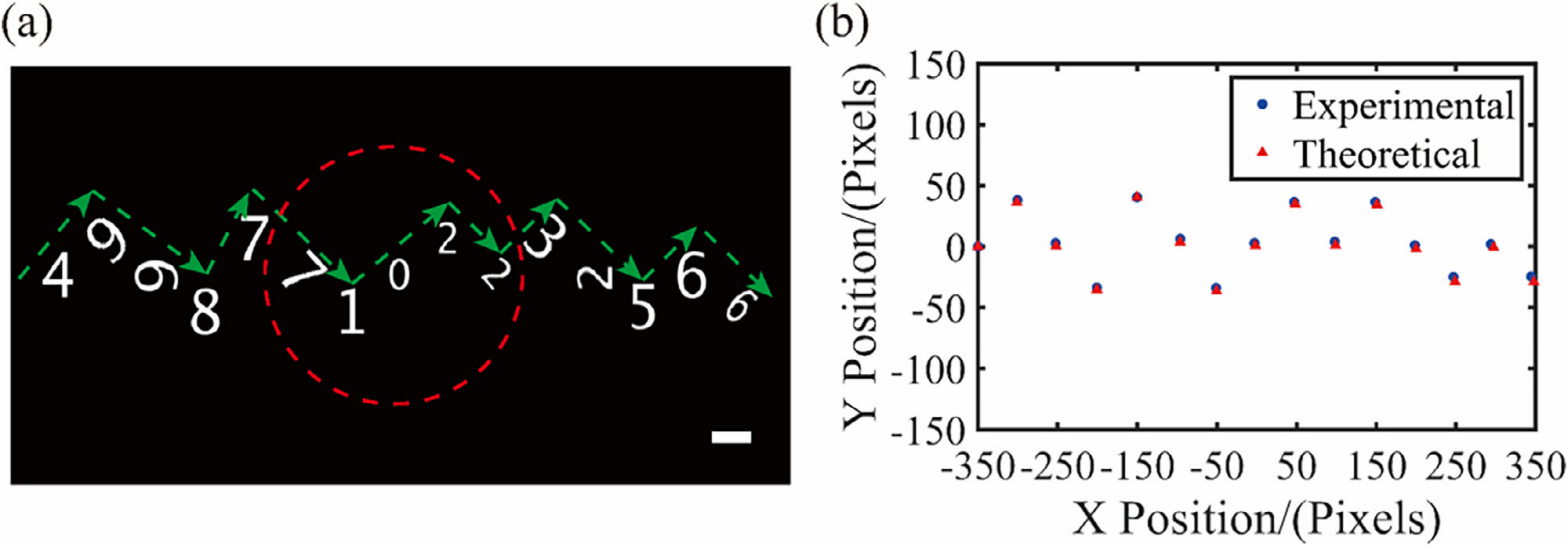
Tracking a moving deformable object inside scattering media. (a) A moving deformable object, hidden inside scattering media. The green arrows indicate the moving direction, and the red‐dashed circle represents the ME region. (b) The comparison of the reconstructed trajectory of the object in (a) (blue dots) and the theoretical trajectory (red triangles). Scale bar: 40 DMD pixels.

To further verify the ability of the SCCLM to track a moving deformable object inside scattering media, an experiment based on the experimental setup in Figure S5b is conducted. In this experiment, the camera exposure time was set to 1.3 s. The light emitted from the LED is first scattered into a speckle pattern by a ground glass diffuser, generating a speckle illumination pattern, and then illuminates the moving deformable object simulated by the DMD. The shape and trajectory of the simulated moving object are arbitrarily changed during motion, and the total movement distance of the object exceeds the ME region, as shown in Figure 4a. The light reflected from the moving object at different moments passes through an iris and a ground glass diffuser, forming a series of speckle patterns. Both ground glass diffusers are 120‐grit. The moving deformable object is illuminated by a speckle pattern. Utilizing our method, the relative centroid displacements of the moving object in the image plane can still be recovered. Its trajectory can be reconstructed by sequentially superimposing the relative centroid displacements of the object plane, as shown in Figure 4b (See Video S3). The reconstructed trajectory almost coincides with the theoretical one, which suggests that even if the moving deformable object is hidden inside the scattering media, its trajectory can still be effectively reconstructed utilizing the SCCLM.

### 2.6 Tracking a Moving Deformable Object Around Corners

**FIGURE 5:**
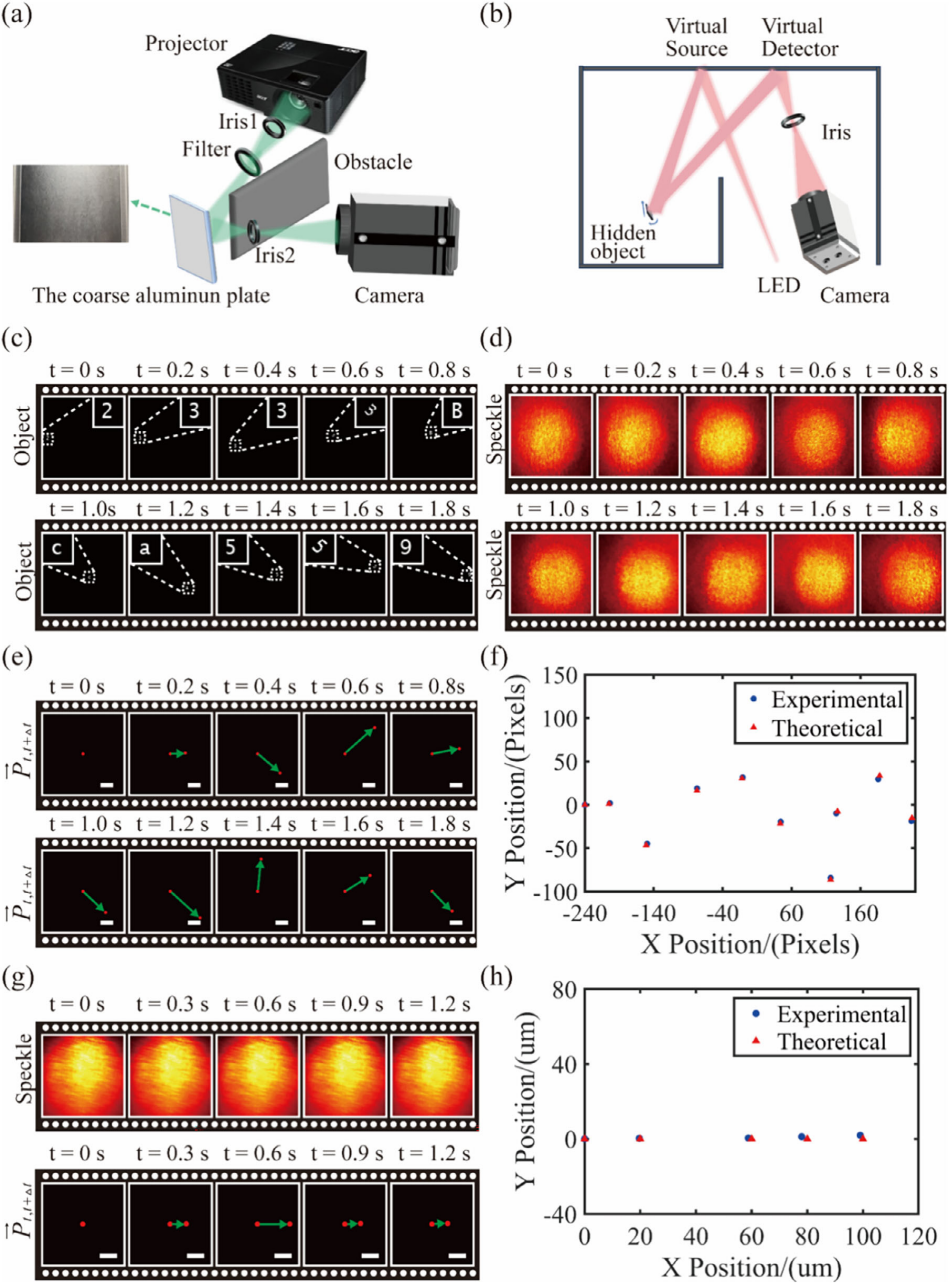
Tracking a moving deformable object around corners. (a) and (b) Experimental setup for tracking a moving object, which is actively emitting light (a) or indirectly illuminated by the LED light source (b). (c) A moving object, hidden around corners, is simulated on the projector in (a), and the speckle patterns are captured by the camera (d). (e) The relative centroid displacements of the moving object (c) in the image plane. Scale bar: 30 camera pixels. (f) The comparison of the theoretical trajectory (red triangles) of the object in (c) and the trajectory reconstructed by the SCCLM (blue dots). (g) The speckle patterns captured from the setup in (b) and the calculated relative centroid displacements at different moments. Scale bar: 20 µm. (h) The comparison of the theoretical trajectory (red triangles) of the reflective object and the trajectory reconstructed by the SCCLM (blue dots).

Non‐line‐of‐sight (NLOS) imaging or tracking enables observation of hidden objects around corners through diffuse reflections from visible surfaces, offering broad potential applications. To verify the capabilities of the SCCLM for tracking a moving deformable object around corners, an experimental setup is designed as shown in Figure 5a (See Section S6 for details). In this experiment, the camera exposure times were set to 0.2 and 0.3 s, respectively. A projector is used to simulate an occluded moving deformable object (Figure 5c), the light emitted from the moving object at different moments is diffusely reflected by the visible surfaces (a coarse aluminum plate), forming the speckle patterns, and collected by the camera (Figure 5d). Through the SCCLM, the relative centroid displacements of the object ${\vec{P}}_{t,t+▵t}$ in the image plane are calculated from the non‐invasively recorded speckle patterns, as shown in Figure 5e, and the trajectory of the object can be reconstructed, as shown in the blue dots in Figure 5f (See Video S4). The results show that the reconstructed trajectory utilizing the SCCLM is well in agreement with the theoretical one.

In addition, we investigate the NLOS tracking scenario depicted in Figure 5b. A physical moving object is indirectly illuminated by an LED light source with a wavelength of 625 nm. The light reflected from the moving object is intercepted by the coarse aluminum plate again and diffusely scattered into a series of speckle patterns (Figure 5g) [40, 41, 42]. This configuration results in the LED light source being scattered twice by the coarse aluminum plate (See Section S6 for details). To simulate a moving object, a reflective object is placed on a translation stage, and the relative centroid displacements of the image plane can be calculated from the recorded speckle patterns. As expected, object tracking can be successfully achieved using the SCCLM, as shown in Figure 5h. Experimental results prove the effectiveness of the SCCLM for NLOS tracking, whether the object is actively emitting light or being illuminated indirectly.

### 2.7 Tracking a Moving Deformable Object in Complex Scenes With Many Objects

**FIGURE 6:**
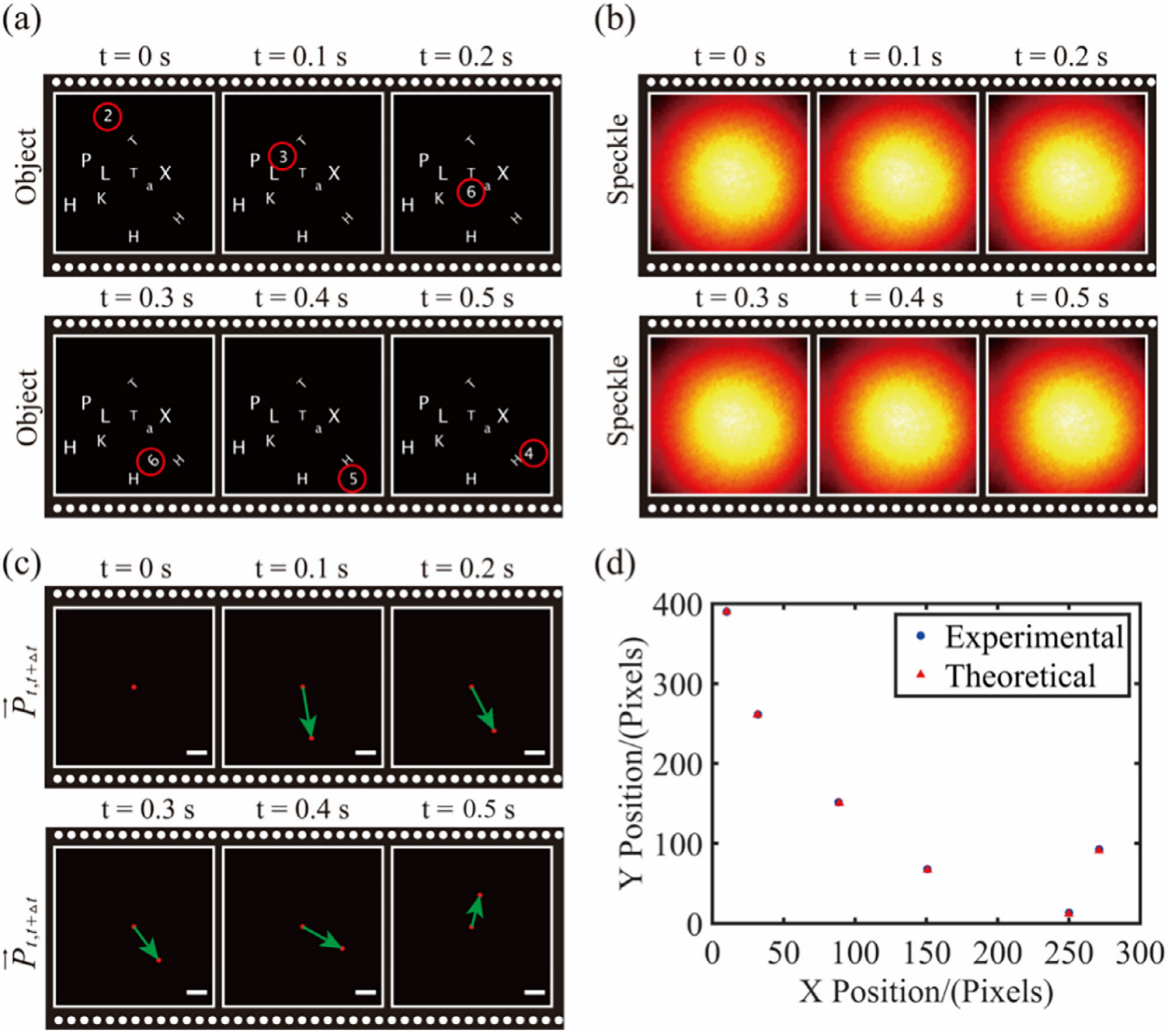
Tracking a moving deformable object in complex scenes with many objects. (a) Ground truth. A moving deformation object over many static objects, hidden behind the scattering media. The red circle indicates the moving object. (b) The speckle patterns captured by the camera. (c) The relative displacements of the moving object. Scale bar: 50 DMD pixels. (d) The comparison of the theoretical trajectory (red triangles) of the object in (a) and the trajectory reconstructed by the SCCLM (blue dots).

To prove the effectiveness of the proposed SCCLM in more complex scene tracking, a moving deformable object over many static objects is designed and shown in Figure 6a. Here, the camera exposure time was set to 0.1 s. The light emitted from the moving deformable object propagates through a ground glass diffuser and produces a series of speckle patterns, as shown in Figure 6b. The speckle pattern of static objects, regarded as background, is extracted by speckle superposition and averaging technology. Then, by subtracting this static background from the camera‐captured speckle patterns, the speckle patterns corresponding to the moving object can be isolated. Further, utilizing the SCCLM to calculate the relative displacements of the moving deformable object at each adjacent moment, as shown in Figure 6c. Finally, the reconstructed trajectory of the moving deformable object in this complex environment is shown in Figure 6d (See Video S6). The results show that the reconstructed trajectory coincides with the theoretical trajectory of the object, which proves the effectiveness of the proposed method for tracking a moving deformable object in complex environments.

## 3 Discussion and Conclusion

Through the above experiments, the SCCLM has been successfully demonstrated for non‐invasive tracking of a moving deformable object behind or inside scattering media and around corners. This should be thanks to the finding that the relative displacements of the moving object are determined by locating the centroid positions of speckle auto‐correlation (SAC) and speckle cross‐correlation (SCC) at different moments, rather than, as the existing methods do [17, 18, 25, 27, 31, 32, 33, 34, 35], conventionally locating the peak positions of SAC and SCC. When the hidden object undergoes only translation without shape changing, the SCC is centrally symmetric, and the peak position of the SCC happens to coincide with its centroid position (Figure S1). In such a special case, the existing method (locating the correlation peak of the SCC) works effectively. In practice, the relative orientation and/or distance between the object and detector normally change randomly, which makes the observed shape of the hidden moving object deform accordingly. In these complex scenarios, the existing methods fail since they can only track an object moving with only translation. However, the SCCLM can solve the problem (See Section S1), because it is not only suitable for tracking a translation object (Figure S1), but also allows the moving object to rotate with translation, scale with translation, or even undergo complete deformation during motion (Figures S2–S4). Therefore, the SCCLM is more general and practical. The existing methods are only a special case of the proposed SCCLM. Furthermore, the SCCLM does not rely on any prior information, which is consistent with our goal of being non‐invasive.

We would like to highlight the effectiveness and robustness of the SCCLM in different scattering scenarios for tracking a moving deformable object. First, the SCCLM successfully reconstructs the trajectories of the moving deformable objects behind static scattering media, including single and double layers of ground glass diffusers (See Figure 2; Figures S6 and S7), where the tracked objects are not only scaling (Figure S6a), rotating and stretching (Figure S6b and S7c), but also deforming into completely different shapes (Figure S7a). Second, experiments were conducted by placing a moving deformable object behind dynamic scattering media with decorrelation. Even so, its trajectory can still be reconstructed as long as the speckle patterns captured at the adjacent moments have some correlation (See Figure 3). Thirdly, the SCCLM also works for tracking a moving deformable object inside scattering media (See Figure 4; Figure S8). Even if the moving deformable object is hidden inside a thicker scattering medium like 22 layers of parafilm, whose optical thickness is approximately 5.2 times the transport mean free path (TMFP) [25], the trajectory reconstructed by the SCCLM agrees with the theoretical trajectory (Figure S12). This suggests our SCCLM still works reliably even in low contrast speckle conditions, such as those encountered in thick scattering media with a thickness of >5 times TMFP. Moreover, the tracking of a deformable object moving around corners can be achieved (See Figure 5), which has the potential to serve as a valuable reference for the practical implementation of NLOS tracking. The reconstruction of objects outside the ME region can be achieved simply by ensuring that the object displacement between adjacent moments remains within the ME region. Another key point is that even when the scene becomes more complex, with many objects present (See Figure 6), the moving deformable object can still be tracked accurately. The experiments, involving the moving object being placed behind the single‐layer ground glass diffuser (Figure S9a2), inside the scattering media (Figure S9b2), and around corners (Figure S9c2), were repeated 10 times. The error bars calculated in all the experimental scenarios do not exceed 2.5 pixels (See Section S7 for more details), demonstrating the robustness of the SCCLM.

In addition, when combined with the phase retrieval algorithm (PRA) [17, 29, 43, 44], the SCCLM can be used not only for tracking but also for imaging the moving deformation object. Interestingly, the speckle patterns for tracking require a lower signal‐to‐noise ratio than those for imaging (See Section S8 for details). In the experiment, while the proposed SCCLM can maintain tracking capability, due to a higher SNR required for the PRA, imaging quality degrades (Figure S10). This is because when the object moves far away from the center of the DMD, the iris obscures partial object information, reducing the speckle pattern SNR. As well as the size of the object further impacts speckle SNR and consequently imaging fidelity, as shown in Figure S10.

The proposed method has the potential to track a deforming fluorescent object with motion hidden inside dynamically scattering media, as well as to help improve the imaging performance of the approach proposed in [34]. It could be expected to be applied in practical biomedical testing, including but not limited to the detection of red blood cells flowing in blood vessels [45], and skin perfusion measurement [46]. Combined with a noninvasive method of obtaining object depth information using the scaling characteristic of the axial ME [47], the SCCLM could be extended to track a moving deformable object in three‐dimensional space behind or inside scattering media and around corners.

In summary, we have proposed and demonstrated a robust method that can non‐invasively track a moving deformable object without any prior information, which is much closer to the practical scenarios. Experimental results suggest it can track a moving deformable object not only behind static and dynamic scattering media but also inside scattering media and around corners, while not being limited by the ME region (See Video S9). Our technique is flexible, robust, and applicable to different scattering media and object‐deforming situations, providing the possibility of applications in tracking complex objects through or inside turbid media [48], and around corners [49].

## Author Contributions

D.W. initiated the idea and derived the theory. W.J. and Y.W. performed the numerical simulations with A.Z.’s participation. D.W., W.J., X.L., and A.Z. designed the initial experiments. W.J. and Y.W. performed the experiments with D.W.’s participation. W.J. and D.W. wrote the manuscript with A.Z.’s contributions. All authors discussed, analyzed, and took responsibility for the results and content of the paper. D.W. and F.L. supervised and contributed to all aspects of the research.

## Conflicts of Interest

The authors declare no competing interests.

## Supporting information




**Supporting File 1**: advs75072‐sup‐0001‐SuppMat.docx

**Supporting File 2**: advs75072‐sup‐0002‐Supplementary Video 1 Tracking a moving deformable object behind a single‐layer ground glass diffuser.mov

**Supporting File 3**: advs75072‐sup‐0003‐Supplementary Video 2 Tracking a moving deformable object behind chicken breast tissue.mov

**Supporting File 4**: advs75072‐sup‐0004‐Supplementary Video 3 Tracking a moving deformable object inside scattering media.mov

**Supporting File 5**: advs75072‐sup‐0005‐Supplementary Video 4 Tracking a moving deformable object around corners.mov

**Supporting File 6**: advs75072‐sup‐0006‐Supplementary Video 5 Tracking a moving deformable object behind two layers of ground glass diffusers.mov

**Supporting File 7**: advs75072‐sup‐0007‐Supplementary Video 6 Complex scenes with many objects.mov

**Supporting File 8**: advs75072‐sup‐0008‐Supplementary Video 7 Tracking and imaging of a moving deformable object behind scattering media.mov

**Supporting File 9**: advs75072‐sup‐0009‐Supplementary Video 8 Tracking a moving deformable object inside scattering media with a thickness of over 5 times the transport mean free path.mov

**Supporting File 10**: advs75072‐sup‐0010‐Supplementary Video 9 for demonstrating of this work.mov

## Data Availability

The data that support the fundings of the study are available from the corresponding author upon request.
