# Supplementary material for: Non‐Invasively Tracking an Arbitrary Deformable Object Through Scattering Media and Around Corners via Speckle Correlations
Source: Adv Sci (Weinh). 2026 Apr 3;13(34):e75072. doi: 10.1002/advs.75072 (PMC13285176; doi:10.1002/advs.75072)
Supplement: Supplementary file 1 — Supporting File 1: advs75072‐sup‐0001‐SuppMat.docx [file ADVS-13-e75072-s003.docx]

Supporting Information

**Title** Non-invasively Tracking an Arbitrary Deformable Object Through Scattering Media and Around Corners via Speckle Correlations

*Author(s)*

*Aiping Zhai^1,3,†^, Wenjing Ji^1,†^,* *Yan Wang^1,†^, Xiyuan Luo^2^, Wenjing Zhao^1,3^, Dong Wang^1,3,4,*^ and Fei Liu^1,2,*^*

*^1^ College of Physics and Optoelectronics Engineering, Taiyuan University of Technology, No. 79 West Main Street, Yingze, 030024, PR China*

*^2^ School of Optoelectronic Engineering, Xidian University, Xi’an, PR China*

*^3^ Shanxi Key Laboratory of Precision Measurement Physics, Taiyuan University of Technology, No. 79 West Main Street, Yingze 030024, PR China*

*^4^ Key Laboratory of Advanced Transducers and Intelligent Control System, Ministry of Education, and Shanxi Province, Taiyuan University of Technology, No. 79 West Main Street, Yingze, 030024, PR China.*

** Corresponding author. Email:* *[wangdong@tyut.edu.cn](mailto:wangdong@tyut.edu.cn), feiliu@xidian.edu.cn*

*† These authors contributed equally to this work.*

**1.** **Comparison of the SCCLM and** **the existing methods for determining the relative displacements of a moving deformable object at adjacent moments**

In this section, the relative displacements of a moving deformable object at adjacent moments calculated by the SCCLM and the existing methods^[1-9]^ are compared. The experiments are designed as follows. Firstly, we set the moving object that only translates (Figure S1). The theoretical relative displacements of the object at the adjacent moments are given in Figure S1(c). The light reflected passes through a 120-grit ground glass diffuser, forming the speckle patterns that are captured by the camera. Then, the relative displacements of the moving deformable object at adjacent moments are calculated using the SCCLM (Figure S1(d)), as well as the existing methods (locating the peak positions of SAC and SCC) (Figure S1(e)), respectively. The results indicate that when an object is only translating during motion, the peak position of SCC happens to be consistent with the centroid position of SCC (Figure S1(f)). This means that when the shape of the moving object is not deformed (only with translation during motion), the existing methods work well, as expected and demonstrated in refs.[1-9]. However, when the moving objects undergo deformation at adjacent moments, such as rotation with translation (Figure S2), scaling with translation (Figure S3), or even deforming into completely different shapes (Figure S4). The relative displacements calculated using the existing methods have large errors, which suggests the existing methods (locating the peak positions of SAC and SCC) normally fail in these complex scenarios. By contrast, the relative displacements can be calculated accurately by the SCCLM (see Figure S2-4), which suggests our SCCLM still works well.


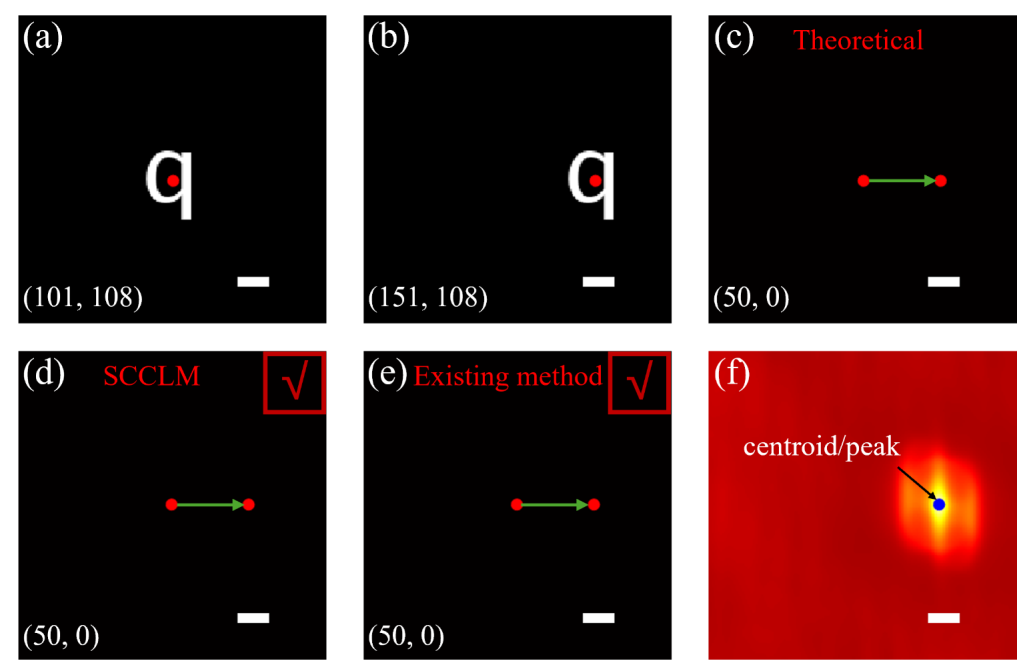


**Figure S1. A moving object undergoes only translation.** (a) and (b) are the states of the hidden object before and after its moving and deforming at adjacent moments. The coordinates at the bottom-left corner indicate the position of the red dot (centroid). (c) The theoretical relative displacements. (d) The relative displacements are calculated with the proposed SCCLM. (e) The relative displacements are calculated with the existing methods (locating the peak positions of SCC). (f) The centroid and peak positions of the object cross-correlation. The blue dots represent the centroid position, and the green dots represent the peak position (The two points coincide). The coordinates at the bottom-left corner represent the displacement vector indicated by the green arrow in panels (c) – (f). Scale bar: 20 DMD pixels.


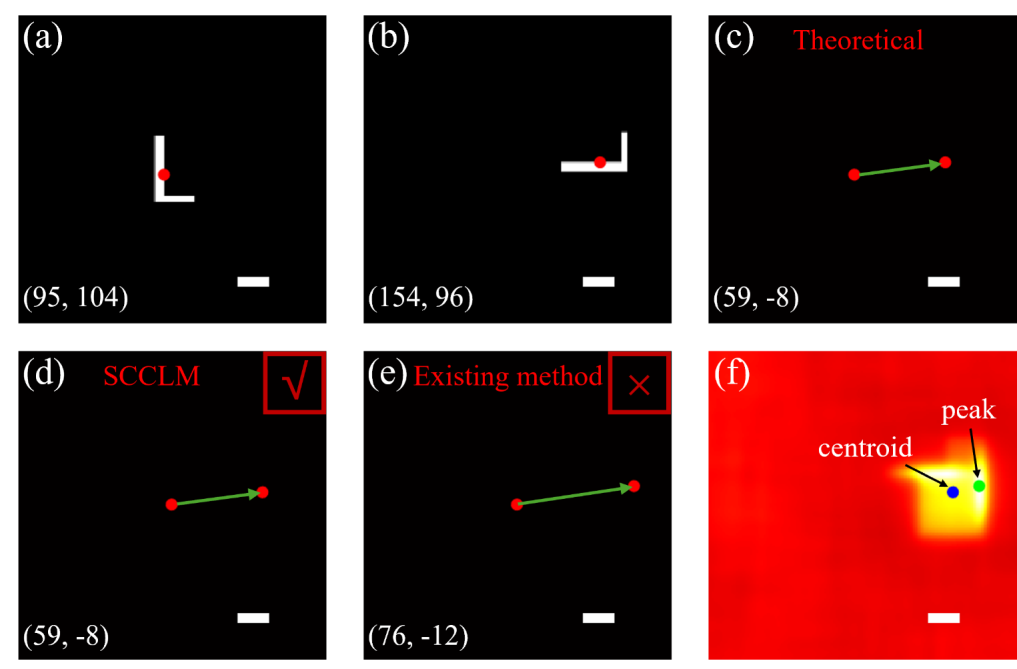


**Figure S2. A moving object undergoes rotation with translation.** (a) and (b) are the states of the hidden object before and after its moving and deforming at adjacent moments. The coordinates at the bottom-left corner indicate the position of the red dot (centroid). (c) The theoretical relative displacements. (d) The relative displacements are calculated with the proposed SCCLM. (e) The relative displacements are calculated with the existing methods (locating the peak positions of SCC). (f) The centroid and peak positions of the object cross-correlation. The blue dot represents the centroid position, and the green dot represents the peak position. The coordinates at the bottom-left corner represent the displacement vector indicated by the green arrow in panels (c) – (f). Scale bar: 20 DMD pixels.


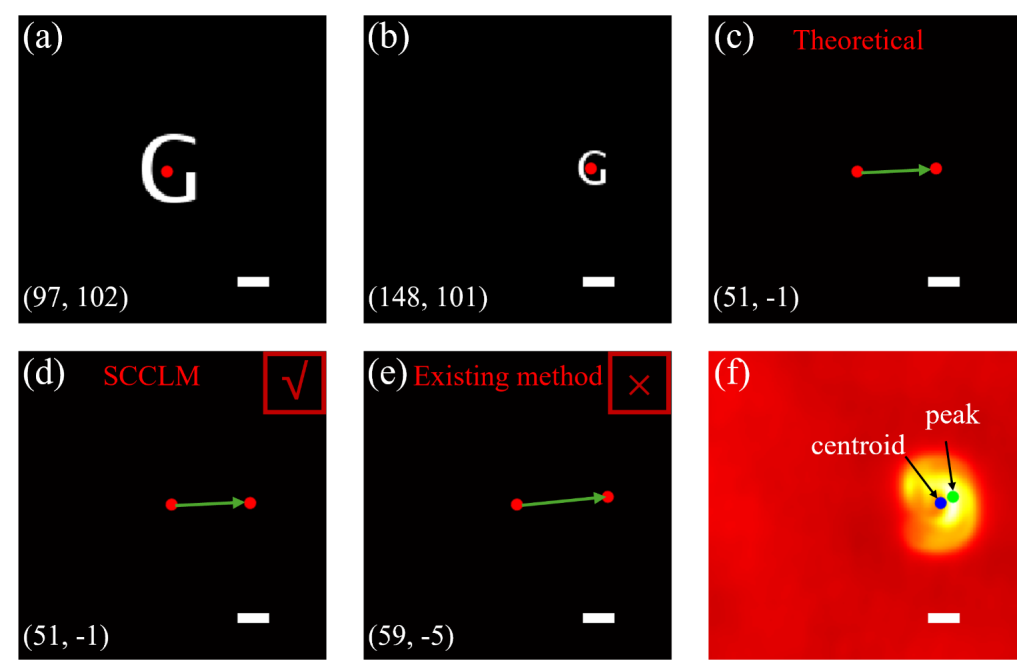


**Figure S3. A moving object undergoes scaling with translation.** (a) and (b) are the states of the hidden object before and after its moving and deforming at adjacent moments. The coordinates at the bottom-left corner indicate the position of the red dot (centroid). (c) The theoretical relative displacements. (d) The relative displacements are calculated with the proposed SCCLM. (e) The relative displacements are calculated with the existing methods (locating the peak positions of SCC). (f) The centroid and peak positions of the object cross-correlation. The blue dot represents the centroid position, and the green dot represents the peak position. The coordinates at the bottom-left corner represent the displacement vector indicated by the green arrow in panels (c) – (f). Scale bar: 20 DMD pixels.

**
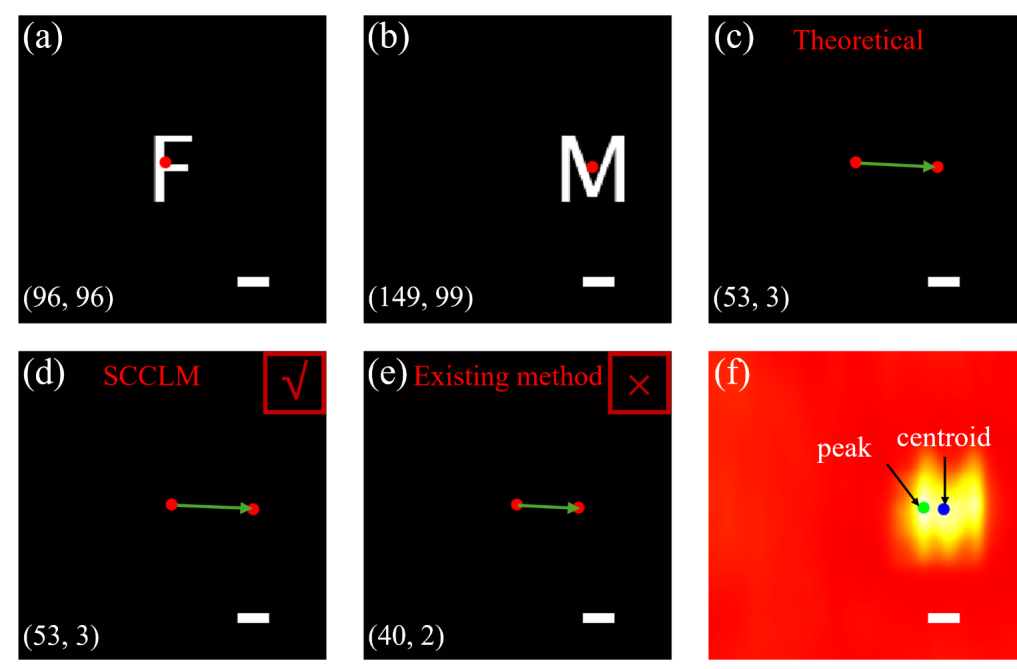
**

**Figure S4. A moving object undergoes complete deformation.** (a) and (b) are the states of the hidden object before and after its moving and deforming at adjacent moments. The coordinates at the bottom-left corner indicate the position of the red dot (centroid). (c) The theoretical relative displacements. (d) The relative displacements are calculated with the proposed SCCLM. (e) The relative displacements are calculated with the existing methods (locating the peak positions of SCC). (f) The centroid and peak positions of the object cross-correlation. The blue dot represents the centroid position, and the green dot represents the peak position. The coordinates at the bottom-left corner represent the displacement vector indicated by the green arrow in panels (c) – (f). Scale bar: 20 DMD pixels.

**2. Experimental setup**

This section describes two experimental setups mentioned in the main text. (I): As shown in Figure S5(a), the setup is used to demonstrate a moving deformable object tracking behind scattering media. The scattering media used in experiments include a single-layer ground glass diffuser (DG10-120, Thorlabs), a double-layer ground glass diffuser (DG10-120, DG10-1500, Thorlabs), and a 1.2 mm thick slice of chicken breast tissue. (II): As shown in Figure S5(b), we added a ground glass diffuser between LED and DMD to simulate tracking a moving deformable object inside scattering media, both scattering media are 120-grit ground glass diffusers (DG10-120, Thorlabs), and other settings are the same as the setup described in Figure S5(a).


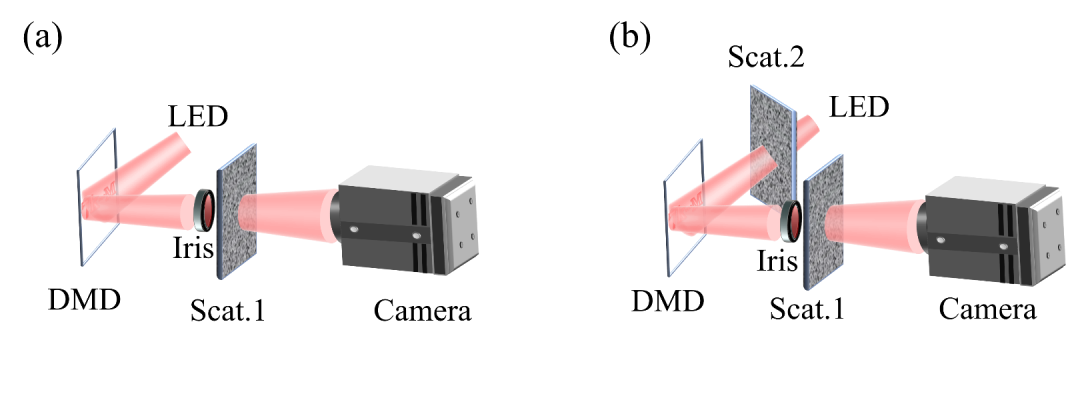


**Figure S5. Experimental setup.** (a) Experimental setup for tracking a moving deformable object behind scattering media. (b) Experimental setup for tracking a moving deformable object inside scattering media.

**3.** **Tracking a moving deformable object behind a single-layer ground glass diffuser.**

We experimentally demonstrate that the SCCLM can track a moving object that is enlarging, rotating, and scaling during motion, utilizing the experimental setup of Figure S5(a). The moving object is hidden behind a single-layer ground glass diffuser (DG10-120, Thorlabs), with a unilateral ME range is approximately 135 DMD pixels. Firstly, a gradually enlarged character ‘*N’* moves along the diagonal direction, as shown in the first row of Figure S6(a). The moving distance of the object within the time interval of $\triangle t=0.3 \text{ s}$ is 57 pixels, while the total moving distance (513 pixels) is 3.8 times the ME region. Utilizing the proposed SCCLM, after capturing a series of speckle patterns (the second row of Figure S6(a)) and processing them with the Gaussian filtering function, the relative centroid displacements $\overrightarrow{P}_{t,t+\triangle t}$ of the moving object in the image plane at different moments can be determined in turn, as shown in the third row of Figure S6(a). Then, the trajectory of the object can be reconstructed by sequentially superimposing the relative centroid displacements $\vec{R}_{t,t+\triangle t}$, denoted as the blue circles in Figure S6(c). The results show that the reconstructed trajectory is consistent with the theoretical one (the red triangles in Figure S6(c)).

Furthermore, as shown in the first row of Figure S6(b), a scenario where a complex object moves laterally while rotating and scaling is simulated. From speckle patterns captured by the camera, the trajectory of the object is reconstructed successfully utilizing the SCCLM, as shown in Figure S6(d). Inevitably, the reconstructed motion trajectory shows consistent trends with the theoretical one, demonstrating the effectiveness of the proposed SCCLM in tracking a moving object with enlarging, rotating, and scaling behind scattering media.

**
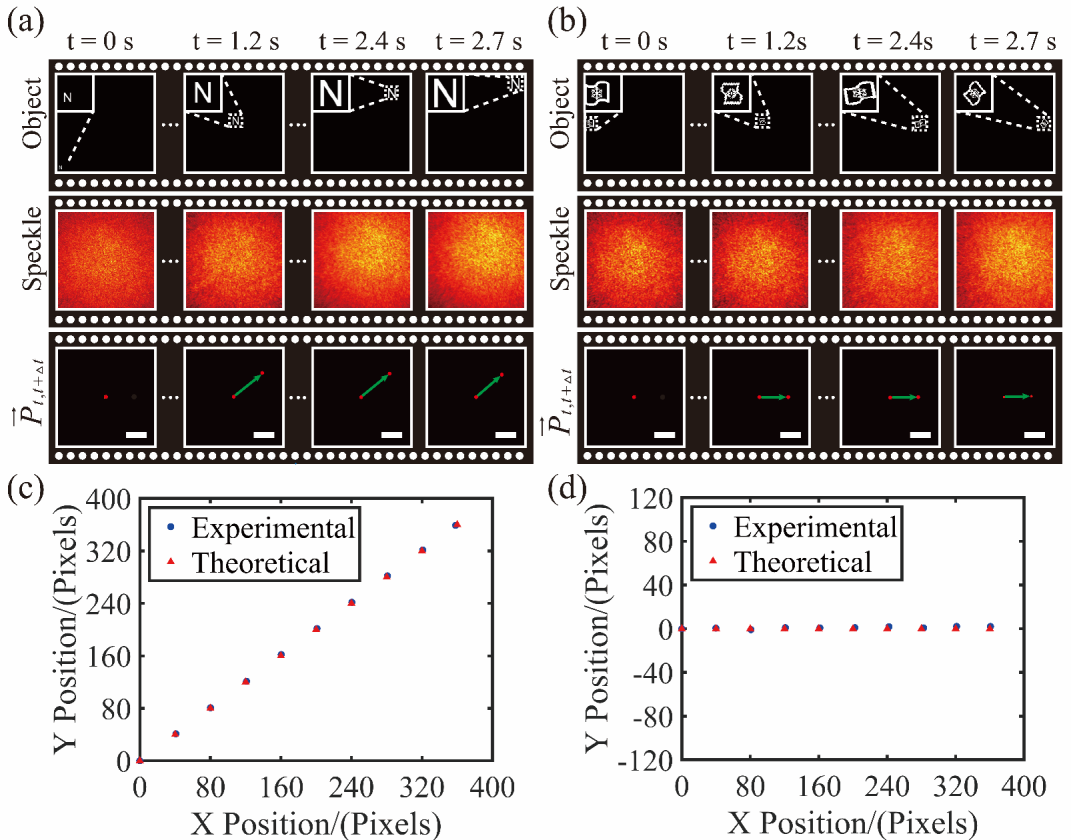
**

**Figure S6. Tracking a moving object behind a single-layer ground glass diffuser.** (a) and (b) In the first row, a moving object gradually enlarges in (a), rotates or stretches in (b). The second row and the third row are the collected speckle patterns and the relative centroid displacements of the image plane at different moments. (c) and (d) The comparison of the theoretical trajectories (red triangles) in (a) and (b) with the trajectories reconstructed by the SCCLM (blue circles), respectively. Scale bar: 20 camera pixels.

**4. Tracking a moving deformable object behind** **two layers of ground glass diffusers.**

To further validate that the SCCLM can also be applicable for tracking a moving deformable object behind stronger scattering media, two ground glass diffusers (DG10-120, DG10-1500, Thorlabs) with a separation distance of 0.17 mm are combined to form a stronger scattering medium, and the unilateral ME range is approximately 93 DMD pixels. We simulate moving deformable objects that are changing into completely different shapes (Figure S7(a)), or rotating and scaling during motion (Figure S7(c)) during each time interval of $\triangle t$. Due to strong scattering characteristics, the speckle intensity decreases, therefore, the time interval $\triangle t$ is set as 0.7 s to enhance the signal-to-noise ratio of the speckle patterns. Utilizing the SCCLM, the relative centroid displacements of the object in the image plane at the adjacent moments are accurately calculated from speckle patterns. Furthermore, even if the object moves 360 DMD pixels from the initial position (i.e. around 3.9 folds of the ME region), the trajectories of the objects are successfully reconstructed by sequentially superimposing the calculated relative centroid displacements in the object plane, as shown in the blue circles in Figure S7(b) and Figure S7(d) (See Supplementary Video 5). Obviously, the SCCLM can effectively track a moving deformable object behind stronger scattering media, without being limited by the ME region.


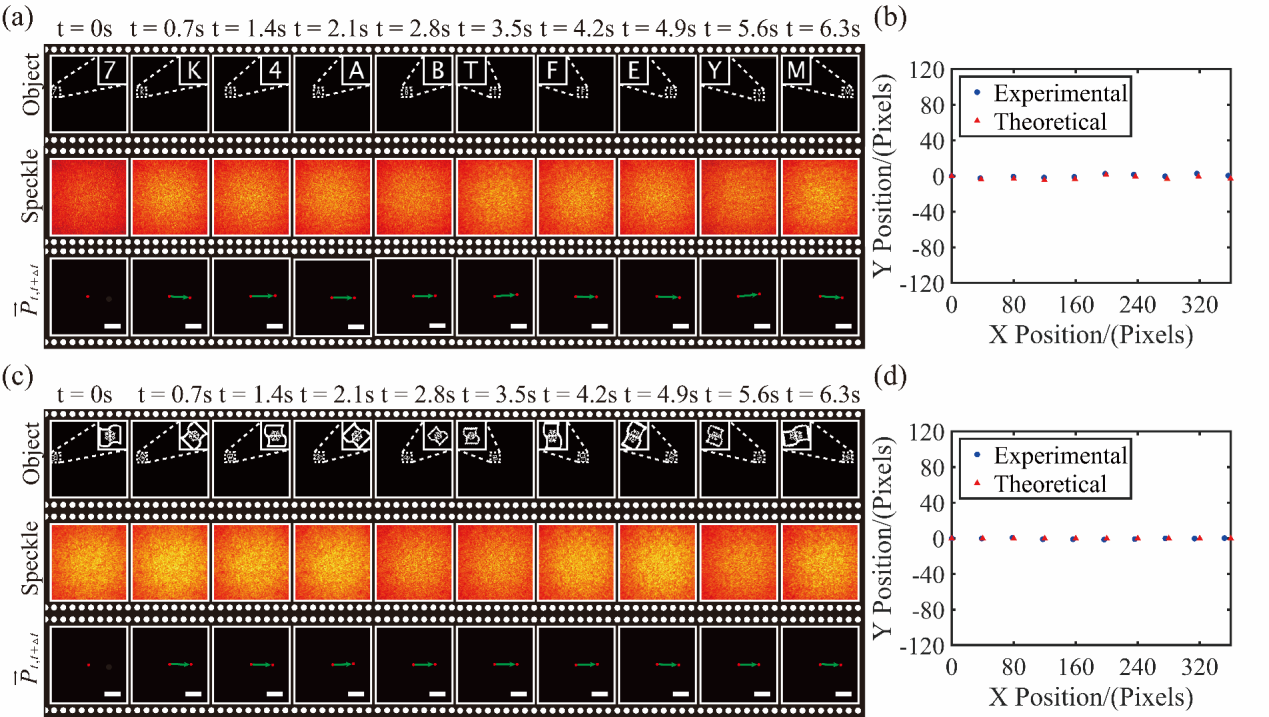


**Figure S7. Tracking a moving deformable object behind two layers of ground glass diffusers.** (a) and (c) In the first row, a moving object changes into completely different shapes in (a), and rotates or stretches in (c). The second row and the third row are the collected speckle patterns and the relative centroid displacements. (b) and (d) The comparison of the theoretical trajectories (red triangles) in (a) and (c) with the trajectories reconstructed by the SCCLM (blue circles), respectively. Scale bar: 20 camera pixels.

**5.** **Tracking a** **deformable object** **moving in** **non-uniform velocity inside scattering media.**

In theory, as long as the adjacent ME regions, in which the moving object is located, have some overlap, the SCCLM can track a moving deformable object effectively, regardless of whether it moves at a uniform speed. To demonstrate it, we conducted experimental validation using the setup as shown in Figure S5(b). Both scattering media are 120-grit ground glass diffusers, and the ME region is approximately 270 DMD pixels. We designed a deforming object with a non-uniform movement step, and the total moving range is 700 DMD pixels, as shown in Figure S8(a). The moving deformable object is simulated with the DMD, and a series of speckle patterns is captured by the camera. Similar to the previous experiments, utilizing the SCCLM, the relative centroid displacements of the moving object are accurately determined, and the trajectory is successfully reconstructed, which is almost consistent with the theoretical trajectory, as shown in Figure S8(b). The experimental results suggest our method still works, meaning that the SCCLM allows tracking an arbitrarily deforming object moving with a non-uniform speed inside scattering media, further demonstrating its robustness beyond uniform motion constraints.

**
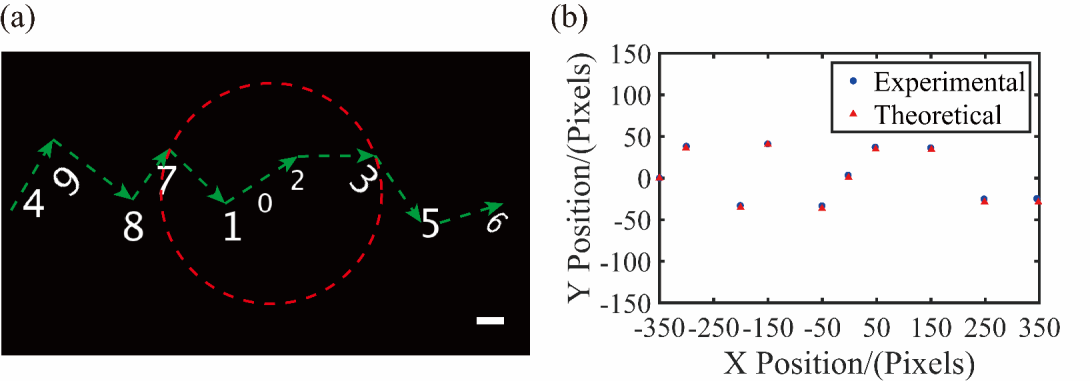
**

**Figure S8. Tracking a deformable object moving in non-uniform velocity inside scattering media.** (a) A moving deformable object, the green arrows indicate the moving direction, and the red-dashed circle represents the ME region. (b) The comparison of the theoretical trajectories (red triangles) in (a) with the trajectories reconstructed by the SCCLM (blue circles). Scale bar: 40 DMD pixels.

**6**. Description of the setup for tracking a moving deformable object around corners.

In this section, two experimental setups for object tracking around corners are described in detail. As shown in Figure 5(a) in the main text, the projector (Acer X1210K) is used to simulate a moving deformable object. Note that the projection lens is removed, and the stray light is removed by placing an iris at the output plane of the projector. An optical filter (MBF532-10, LBTEK) is used to narrow the optical spectrum. Light carrying information about the moving object is diffusely reflected by a visible surface (The coarse aluminum plate), and a series of speckle patterns is recorded by the camera (Andor Zyla 5.5, 2560 × 2160 pixels, pixel size 6.5 µm). Another 3.5 mm iris is placed between the visible surface and the camera to adjust the resolution of the captured speckle patterns.

Furthermore, another experimental setup for object tracking around corners is shown in Figure 5(b) in the main text, where the object is indirectly illuminated by diffuse light from the visible surface (The coarse aluminum plate). A hollow object (horizontal line) is placed on a 3D translation stage to simulate a moving object, and a reflector is placed behind it to create a reflective object. A 625 nm LED illuminates a spot on the coarse aluminum plate (the virtual source), scattering light toward the obscured moving object. The light incident on the moving deformable object is reflected to the aluminum plate (the virtual detector), forming speckle patterns captured by the camera. A 5 mm iris is placed in front of the camera to adjust the resolution of the captured speckle patterns. The distance *u* from the hidden object to the aluminum plate is 130 mm, and the distance *v* from the aluminum plate to the camera is 102 mm.

**7. Error analysis in recovering the trajectory of a moving deformable object using the proposed SCCLM.**

To determine the robustness of the proposed SCCLM, repeated experiments are conducted to quantitatively analyze the error in recovering the trajectory of a moving deformable object, hidden behind or inside scattering media, as well as around corners. First, when a moving deformable object is hidden behind a single-layer ground glass diffuser, we repeated the experiment shown in Figure 2(e) and Figure 7(a1) 10 times and reconstructed the trajectories separately each time. Next, we calculated the average trajectory of the 10 trajectories and the error bars. The results show that the maximum error bar is only 0.4 DMD pixels, as shown in the enlarged area of Figure S9(a2). Secondly, utilizing the same method as above, we calculated the average trajectory and the error bars for 10 repeated experiments, as shown in Figure 4(a) and Figure S9(b1), in which the moving deformable object is hidden inside the scattering media. The maximum error bar is approximately 1 DMD pixels, as shown in the enlarged area of Figure S9(b2). Finally, when the moving deformable object is hidden around corners (Figure 5(c) and Figure S9(c1)), all the error bars of 10 repeated experiments are within a range of 2 pixels, as shown in the enlarged area of Figure S9(c2). The experimental results show the reliability of the proposed SCCLM in different environments.

**
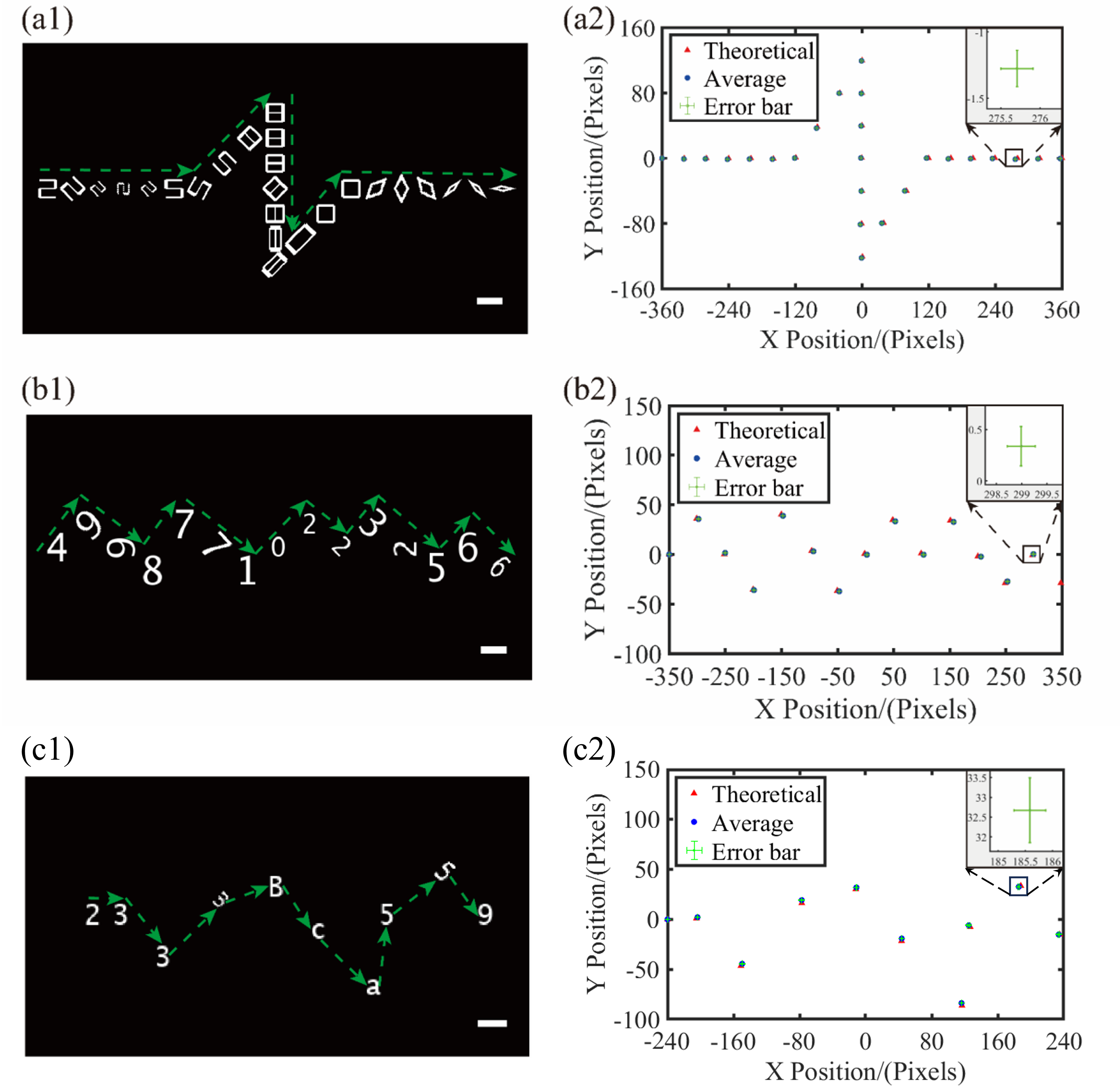
**

**Figure S9. Error analysis in recovering the trajectory of a moving deformable object using the proposed SCCLM.** (a1) A moving deformable object hidden behind a single-layer ground glass diffuser. (a2) The red triangles and the blue circles indicate the theoretical trajectory and the average trajectory of the object in (a1), respectively. Green indicates error bars, and the enlarged area indicates the maximum error bar. (b1) A moving deformable object inside scattering media. (b2) The red triangles and the blue circles indicate the theoretical trajectory and the average trajectory of the object in (b1), and the other descriptions are the same as in (a2). (c1) A moving deformable object around corners. (c2) The red triangles and the blue circles indicate the theoretical trajectory and the average trajectory of the object in (c1), and the other descriptions are the same as in (a2). Scale bar: 40 DMD pixels.

**8.** **Imaging and tracking of a moving deformable object hidden behind scattering media.**

To realize the tracking and imaging of a moving deformable object, we guide the following experiment. Firstly, a moving deformable object hidden behind the scattering media is shown in Figure 2(e) and Figure S10(a). After capturing the speckle patterns of the moving object at different moments, utilize the phase retrieval algorithm to image the moving object. Combined with the relative displacements determined by SCCLM, the tracking and imaging of the moving deformable object can be realized, as shown in Figure S10(b). From the imaging results, it is found that the imaging effect at some moments is poor, although the relative displacements can be calculated accurately. A possible explanation is that a higher signal-to-noise ratio for the speckle patterns is required to image an object using a phase retrieval algorithm. However, when the size of the moving object at some moments is small, or when the object moves far away from the center of the DMD, part of the information is obscured by the iris in Figure S5(a), resulting in the signal-to-noise ratio of speckle patterns captured by the camera is reduced. To prove the above explanation, a moving deformable object of a large size is designed, as shown in Figure S10(c). The moving object at different moments is loaded onto the DMD sequentially, and the speckle patterns are acquired by the camera. Combined with the phase retrieval algorithm and SCCLM, the imaging and tracking of a moving deformation object hidden behind the scattering media can be realized, as shown in Figure S10(d). It can be seen that the imaging effect of the moving object is improved with the increase of the speckle signal-to-noise ratio. In conclusion, as long as the speckle patterns have a sufficient signal-to-noise ratio, the SCCLM proposed in this manuscript can be combined with the phase retrieval algorithm to achieve the imaging and tracking of the moving deformation object.

***
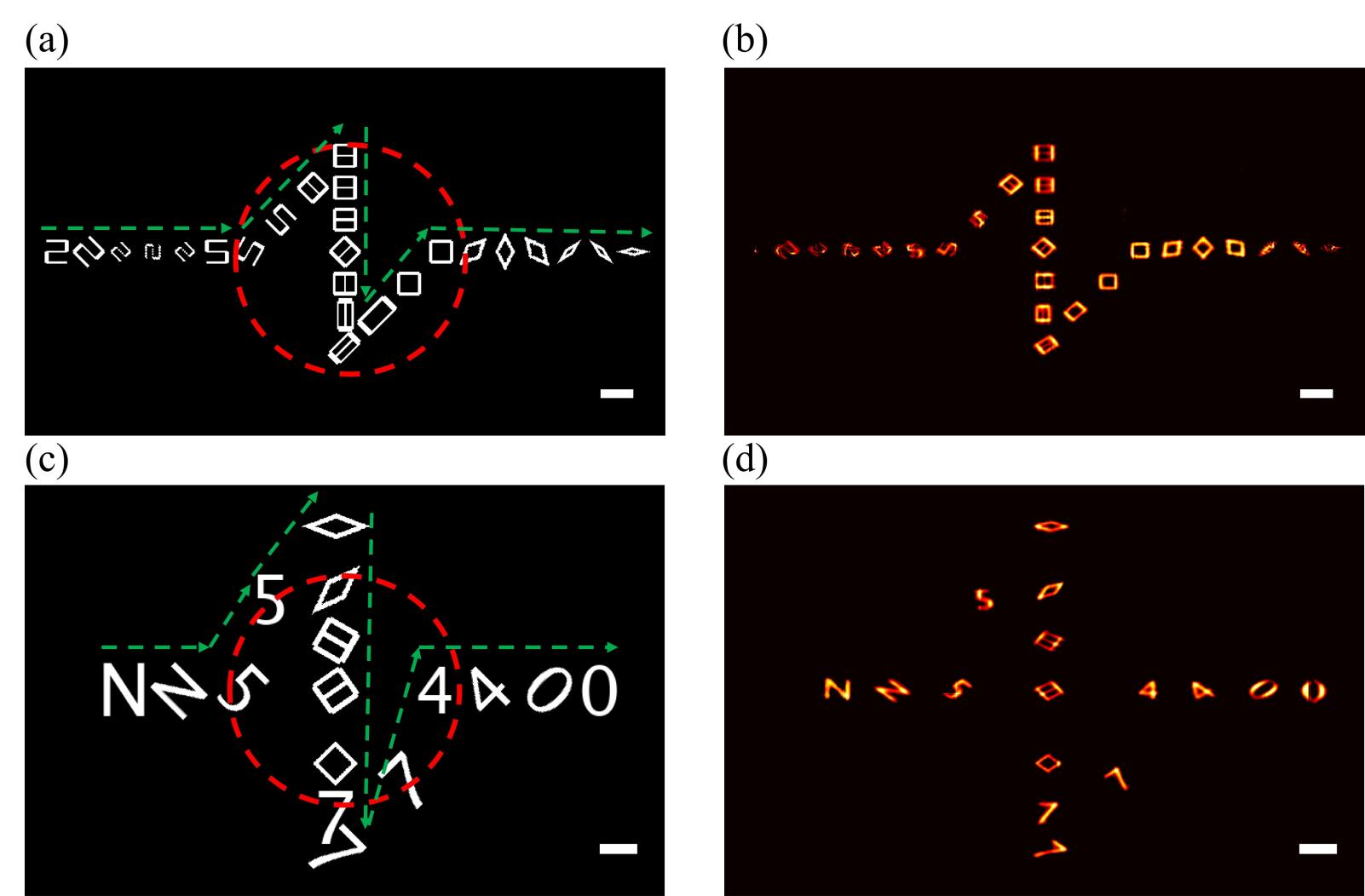
***

**Figure S10. Tracking and imaging of a moving deformable object behind scattering media.** (a) The same moving deformable object as Figure 2e in the text. (b) Imaging and tracking results of the moving deformable object in (a). (c) A moving deformable object is designed with a large size. (d) Imaging and tracking results of the moving deformable object in (c). The green arrows indicate the moving direction, and the red-dashed circle represents the ME region. Scale bar: 40 DMD pixels.

**9. Relationship between the cross-correlation of the object and the cross-correlation of the speckle at adjacent moments.**

To investigate the relationship between speckle cross-correlation and object cross-correlation, we simulated four different types of object motion: only translation (Figure S11(a1) - (d1)), rotation with translation (Figure S11(a2) - (d2)), scaling with translation (Figure S11(a3) - (d3), and complete deformation (Figure S11(a4) - (d4)). In each case, we obtained the corresponding speckle patterns of the moving object at times $t$and (Figure S11(a1) - (a4)) and Figure S11(b1) - (b4)), and subsequently calculated the speckle cross-correlation (Figure S11(c1) - (c4)) and the object cross-correlation (Figure S11(d1) - (d4)). The results show that, regardless of how the object moves, there is a clear mathematical relationship between the speckle cross-correlation and the object cross-correlation, differing only by an additional constant background term. This relationship can be expressed as:

$$I_{t}(u,v)★I_{t+\triangle t}(u,v)\text{=}O_{t}(u,v)★O_{t+\triangle t}(u,v)+B$$

where $B$ is the additional constant background term for the speckle cross-correlation.


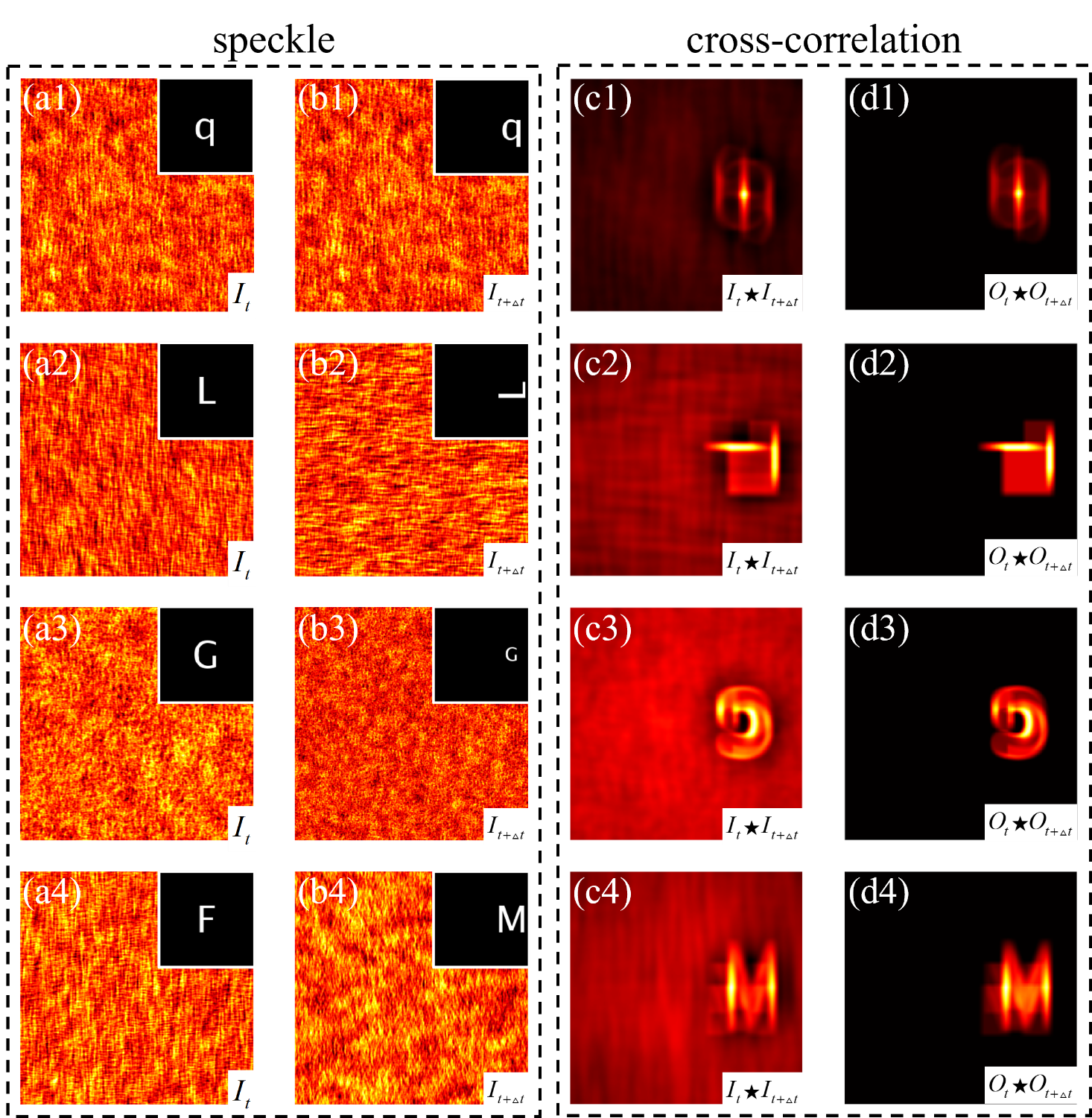


**Figure S11. Relationship between the cross-correlation of the object and the cross-correlation of the speckle at adjacent moments.** (a1) - (a4) and (b1) - (b4) describe speckle patterns of the moving objects (translation, rotation with translation, scaling with translation, and complete deformation during motion) obtained at time $t$ and $t+\triangle t$, respectively. (c1) - (c4) The cross-correlation speckle patterns and speckle patterns . (d1) - (d4) The cross-correlation between object $O_{t}$ and object $O_{t+\triangle t}$.

**10. Tracking a moving deformable object inside scattering media with a thickness of over 5 times the transport mean free path.**

To demonstrate the performance of the proposed SCCLM method in scattering media with large optical thickness, an experiment was conducted using the setup illustrated in Figure S5(b). In this experiment, a moving deformable object depicted in Figure 12(a) was hidden inside 22 layers of parafilm, corresponding to an optical thickness of 5.2 times the transport mean free path (TMFP), which has been measured by the authors in ref.[3]. As presented in Figure S12(b), the trajectory reconstructed by the SCCLM agrees with the theoretical trajectory. This suggests our SCCLM still works reliably even in low contrast speckle conditions, such as those encountered in thick scattering media with a thickness of >5 times TMFP.

**
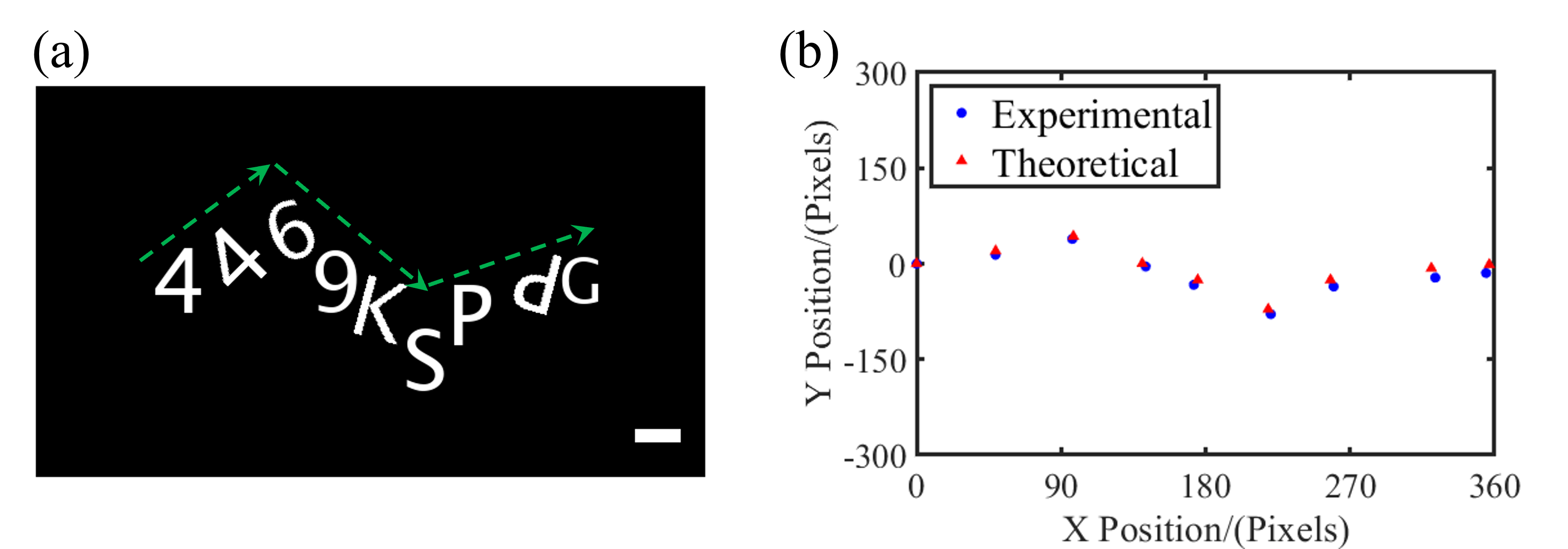
**

**Figure S12. Tracking a moving deformable object inside scattering media with a thickness of over 5 times transport mean free path.** (a) A moving deformable object, hidden inside the parafilm. The green arrows indicate the moving direction. (b) Reconstructed trajectory of the object (blue dots) compared with the theoretical trajectory (red triangles). Scale bar: 40 DMD pixels.

**Supplementary Video 1:** Tracking a moving deformable object behind a single-layer ground glass diffuser

**Supplementary Video 2:** Tracking a moving deformable object behind chicken breast tissue

**Supplementary Video 3:** Tracking a moving deformable object inside scattering media

**Supplementary Video 4:** Tracking a moving deformable object around corners

**Supplementary Video 5:** Tracking a moving deformable object behind two layers of ground glass diffusers

**Supplementary Video 6:** Complex scenes with many objects

**Supplementary Video 7:** Tracking and imaging of a moving deformable object behind scattering media

**Supplementary Video 8：**Tracking a moving deformable object inside scattering media with a thickness of over 5 times the transport mean free path

**References**

1. Wang, D., Sahoo, S. K., Zhu, X., Adamo, G., Dang, C. Non-invasive super-resolution imaging through dynamic scattering media. *Nat. Commun.* **12**, 3150 (2021).

2. Zhang, T., Wang, X., Zhao, W., Zhai, A., Dang, C., Wang, D. Noninvasive imaging through scattering media with enlarged FOV using psf estimations and correlations. *Adv. Photonics Res.* **4**, 2300100 (2023).

3. Shi, Y., Sheng, W., Fu, Y., Liu, Y. Overlapping speckle correlation algorithm for high-resolution imaging and tracking of objects in unknown scattering media. *Nat. Commun.* **14**, 7742 (2023).

4. Jauregui-Sánchez, Y., Penketh, H., Bertolotti, J. Tracking moving objects through scattering media via speckle correlations. *Nat. Commun.* **13**, 5779 (2022).

5. Boniface, A., Dong, J., Gigan, S. Non-invasive focusing and imaging in scattering media with a fluorescence-based transmission matrix. *Nat. Commun.* **11**, 6154 (2020).

6. Guo, C., Liu, J., Wu, T., Zhu, L., Shao, X. Tracking moving targets behind a scattering medium via speckle correlation. *Appl. Opt.* **57**, 905-913 (2018).

7. Xu, Q., Zhao, J., Sun, C., Du, L., Sun, B., Li, X. Edge memory effect for hidden object tracking. *Opt. Lasers Eng.* **151**, 106928 (2022).

8. Huang, Y., Zhao, W., Zhai, A., Wang, D. Noninvasive Fluorescence Imaging Through Scattering Media Beyond Memory Effect via Speckle Correlations. *Laser Photonics Rev.*, 2301020.

9. Zhang, J.*, et al.* Multi-target object scattering imaging with intensity correlation of structured illumination. *Opt. Lett.* **48**, 1486-1489 (2023).
